# Potentially effective drugs for the treatment of COVID-19 or MIS-C in children: a systematic review

**DOI:** 10.1007/s00431-022-04388-w

**Published:** 2022-02-22

**Authors:** Zijun Wang, Siya Zhao, Yuyi Tang, Zhili Wang, Qianling Shi, Xiangyang Dang, Lidan Gan, Shuai Peng, Weiguo Li, Qi Zhou, Qinyuan Li, Joy James Mafiana, Rafael González Cortés, Zhengxiu Luo, Enmei Liu, Yaolong Chen

**Affiliations:** 1 Evidence-Based Medicine Center, School of Basic Medical Sciences, Lanzhou University, Lanzhou, China; 2 Lanzhou University Institute of Health Data Science, Lanzhou, China; 3 School of Public Health, Lanzhou University, Lanzhou, China; 4 Department of Respiratory Medicine, Children’s Hospital of Chongqing Medical University, Chongqing, China; 5 National Clinical Research Center for Child Health and Disorders, Ministry of Education Key Laboratory of Child Development and Disorders, China International Science and Technology Cooperation Base of Child Development and Critical Disorders, Children’s Hospital of Chongqing Medical University, Chongqing, China; 6 Chongqing Key Laboratory of Pediatrics, Chongqing, China; 7 The First School of Clinical Medicine, Lanzhou University, Lanzhou, China; 8 Paediatric Intensive Care Unit, Hospital General Universitario Gregorio Marañón, Calle Doctor Castelo 47, 28007 Madrid, Spain; 9 WHO Collaborating Centre for Guideline Implementation and Knowledge Translation, Lanzhou, China; 10 Lanzhou University GRADE Center, Lanzhou, China

**Keywords:** Children, COVID-19, MIS-C, Glucocorticoids, Intravenous immunoglobulin, Remdesivir

## Abstract

The purpose of this systematic review is to evaluate the efficacy and safety of using potential drugs: remdesivir and glucocorticoid in treating children and adolescents with COVID-19 and intravenous immunoglobulin (IVIG) in treating MIS-C. We searched seven databases, three preprint platform, ClinicalTrials.gov, and Google from December 1, 2019, to August 5, 2021, to collect evidence of remdesivir, glucocorticoid, and IVIG which were used in children and adolescents with COVID-19 or MIS-C. A total of nine cohort studies and one case series study were included in this systematic review. In terms of remdesivir, the meta-analysis of single-arm cohort studies have shown that after the treatment, 54.7% (95%CI, 10.3 to 99.1%) experienced adverse events, 5.6% (95%CI, 1.2 to 10.1%) died, and 27.0% (95%CI, 0 to 73.0%) needed extracorporeal membrane oxygenation or invasive mechanical ventilation. As for glucocorticoids, the results of the meta-analysis showed that the fixed-effect summary odds ratio for the association with mortality was 2.79 (95%CI, 0.13 to 60.87), and the mechanical ventilation rate was 3.12 (95%CI, 0.80 to 12.08) for glucocorticoids compared with the control group. In terms of IVIG, most of the included cohort studies showed that for MIS-C patients with more severe clinical symptoms, IVIG combined with methylprednisolone could achieve better clinical efficacy than IVIG alone.

*Conclusions*: Overall, the current evidence in the included studies is insignificant and of low quality. It is recommended to conduct high-quality randomized controlled trials of remdesivir, glucocorticoids, and IVIG in children and adolescents with COVID-19 or MIS-C to provide substantial evidence for the development of guidelines.

**Table:** 

**What is Known:** *• The efficacy and safety of using potential drugs such as remdesivir, glucocorticoid, and intravenous immunoglobulin (IVIG) in treating children and adolescents with COVID-19/MIS-C are unclear.*	
**What is New:** *• Overall, the current evidence cannot adequately demonstrate the effectiveness and safety of using remdesivir, glucocorticoids, and IVIG in treating children and adolescents with COVID-19 or MIS-C.* *• We are calling for the publication of high-quality clinical trials and provide substantial evidence for the development of guidelines.*

**Supplementary Information:**

The online version contains supplementary material available at 10.1007/s00431-022-04388-w.

## Introduction

It is over a year and a half since the outbreak of coronavirus disease 2019 (COVID-19), and during this period, studies on COVID-19 are continuously emerging [1, 2]. Researchers have paid much attention to drug therapy all the time [3]. Recent studies on COVID-19 drugs and clinical guidelines have focused primarily on adult patients but less attention on children and adolescents. Although children and adolescents with COVID-19 seem less susceptible and have milder symptoms once infected, they are also at risks of advancing to severe stages [4]. Children and adults are known to have physiological differences [5]; thus, many effective COVID-19 drugs for adults may not suitable for children. Among these drugs, remdesivir, glucocorticoids, and intravenous immunoglobulin (IVIG) in children and adolescents have been controversial.

Remdesivir is a broad-spectrum antiviral medication that can integrate into the RNA strand of severe acute respiratory syndrome coronavirus-2 (SARS-CoV-2) and prematurely terminate the ribonucleic acid (RNA) replication process [6]. The World Health Organization (WHO) living guideline for COVID-19 [7] and the US guideline for pediatric COVID-19 [8] have contradicting recommendations for the treatment of children and adolescents, based on evidence from randomized controlled trials of adults.

Glucocorticoids are the most widely used and effective anti-inflammatory and immunosuppressive agents in clinical practice. They have the potential to reduce the severity of lung inflammation in patients with severe COVID-19 [9, 10]. Glucocorticoids are affordable, easy to administer, and readily available globally [11]. The WHO living guidance on glucocorticoids for COVID-19 [12] recommends systemic glucocorticoids to treat adult patients with severe COVID-19. However, the living guidance further suggests that the recommendation is underrepresented in children and adolescents with COVID-19.

IVIG is a recommended first-line therapy for Kawasaki disease because it produces anti-inflammatory effect, which reduces coronary artery abnormalities and myocarditis in patients with Kawasaki disease [13]. MIS-C (multi-system inflammatory syndrome in children) is a newly defined clinical syndrome associated with SARS-CoV-2 infection characterized by fever, systemic inflammation, and multiple organ dysfunction. Several case definitions of this novel inflammatory condition have been published by the WHO [14], the US Center for Disease Control and Prevention (CDC) [15], and the UK of Great Britain Royal College of Pediatrics and Child Health (RCPCH) [16]. The clinical features of MIS-C are similar to those of Kawasaki disease, toxic shock syndrome, sepsis, and macrophage activation syndrome [17]. Hence, the application of IVIG in the treatment of MIS-C is a potential drug choice [18].

As mentioned above, the efficacy and safety of these drugs in the treatment of COVID-19 or MIS-C in children are still unclear because of the lack of large controlled clinical trials. Although cases series or other studies are not reliable evidence to support, but we still need to know the status of the researches. Therefore, we aimed to determine the efficacy and safety of using (1) remdesivir in treating children and adolescents with COVID-19, (2) glucocorticoids in treating children and adolescents with severe COVID-19, and (3) IVIG in treating children and adolescents with MIS-C based on existing researches. Furthermore, provide evidence to support the development of clinical practice guidelines.

## Methods

Six researchers in three groups of two (Group 1: Zijun Wang, Qianling Shi; Group 2: Siya Zhao, Qi Zhou; Group 3: Yuyi Tang, Weiguo Li) retrieved and selected studies, extracted and analyzed data, and interpreted the results. Group 1 focused on remdesivir in treating children and adolescents with COVID-19, Group 2 focused on glucocorticoids in treating children and adolescents with severe COVID-19, and Group 3 focused on IVIG in treating children and adolescents with MIS-C. We reported our study in accordance to the Preferred Reporting Items for Systematic Reviews and Meta Analyses (PRISMA) 2020 guidelines. [19] (Supplementary File 1).

### Search strategy

Two researchers in each group independently searched for literature using MEDLINE (via PubMed), Web of Science, the Cochrane library, China Biology Medicine (CBM), China National Knowledge Infrastructure (CNKI), Wanfang Data, and WHO COVID-19 database (https://search.bvsalud.org/global-literature-on-novel-coronavirus-2019-ncov/), ClinicalTrials.gov (https://clinicaltrials.gov/), MedRxiv (https://www.medrxiv.org/), BioRxiv (https://www.biorxiv.org/), SSRN (https://www.ssrn.com/index.cfm/en/), and Google. The electronic search was supplemented by manually examining the reference lists of the identified studies. In addition, emails were sent to the authors of studies to request available data that may be useful for our systematic review. The data search was from December 2019 to August 2021 without language limitations.

The researchers in groups 1, 2, and 3 used “remdesivir,” “corticosteroids,” and “intravenous immunoglobulin,” and its derivatives as retrieval terms, respectively. The terms were also combined with “COVID-19” and its derivatives using “AND.” For question 3, “MIS-C” and its derivatives were added as retrieval terms and combined with “AND” to improve the accuracy of the search. The search strategy can be found in Supplementary File 2.

### Eligibility criteria

#### Inclusion criteria

Clinical question 1 (remdesivir).

Randomized controlled trials, non-randomized controlled trials, cohort studies, and case series of children and adolescents (≤ 18-year-old) with COVID-19 treated with remdesivir.

Clinical question 2 (glucocorticoids).

Randomized controlled trials, non-randomized controlled trials, cohort studies, and case series of COVID-19 children and adolescents (≤ 18 year) patients treated with glucocorticoids.

Clinical question 3 (IVIG).

1. The study population must meet the diagnostic criteria for MIS-C (WHO [20], CDC [21], or The Royal College of Paediatrics and Child Health [22]), and the included patients were not restricted by age, gender, disease course, race, region, and other factors.
2. The interventions/exposure included IVIG (intravenous immunoglobulin) vs. placebo or other treatment, or IVIG combined with other treatment vs. basic treatment.
3. Inclusion of studies was not restricted by the type of publication.

#### Exclusion criteria

Clinical question 1 (remdesivir).

1. Studies that failed to show the efficacy of remdesivir.
2. Case series that remdesivir was not administered to all the patients or subgroup comparison of remdesivir was unavailable.
3. Full text not available (example, studies inaccessible for download, conference abstract).
4. Duplications.

Clinical question 2 (glucocorticoids).

1. Studies that failed to show the efficacy of glucocorticoids.
2. Case series that glucocorticoid was not administered to all the patients or subgroup comparison of glucocorticoid was unavailable.
3. Full text not available (example, studies inaccessible for download, conference abstract).
4. Duplications.

Clinical question 3 (IVIG).

1. In vitro studies (example, animal experiments, in vitro experiments).
2. Full text not available (e.g., studies inaccessible for download, conference abstract).
3. Duplications.

### Study selection

Two researchers in each group independently screened literature using the EndNote citation management software, and any disagreements were resolved by discussion. Before the formal screening process, researchers in each group randomly selected 50 studies to undertake a pilot study selection and ensure consistency in understanding the inclusion and exclusion criteria. Researchers used the inclusions and exclusions criteria first to screen the studies’ title and abstracts and excluded irrelevant literature. Then, the full text of the literature was reviewed to include the final eligible studies. Finally, the reasons for exclusion were recorded. The details of study selection are shown in the PRISMA 2020 flow diagram (Supplementary File 3).

### Data extraction

Two researchers in each group extracted data independently in pairs, using a predefined data extraction form. Disagreements regarding the data extraction were resolved by discussion. The following information was extracted from the included studies: (1) baseline characteristics: author, year of publication, country, journal, number of included patients, gender, age, study design, and medication taken for COVID-19; (2) data extracted for clinical question 1: adverse events, severe adverse events, mortality, extracorporeal membrane oxygenation (ECMO) or invasive mechanical ventilation (IMV), length of hospital stay, hospital discharge, and symptom duration; (3) data extracted for clinical question 2: mortality, mechanical ventilation, and duration of pediatric intensive care unit (PICU) admission; and (4) data extracted for clinical question 3: number of patients who had treatment failure or secondary acute left ventricular dysfunction, number of patients who needed second-line treatment or hemodynamic support, the duration of PICU stay, isovolumic relaxation time, and the time to recovery of left ventricle ejection, cardiovascular dysfunction, left ventricular dysfunction, shock resulting in vasopressor use, use of adjunctive therapy, receipt of inotropic support or mechanical ventilation, reduction in the score for disease severity, and rate of c-reactive protein (CRP) levels less than 60 mg/L by day 3.

For dichotomous variables, the data of the number of events and the total of events were extracted. For continuous variables, mean, standard deviation, and the number of included patients were extracted. The median, quartile, maximum values, and minimum values were converted into mean and standard deviation using methods of estimating math [23]. Studies were excluded from the meta-analysis if the primary data was unavailable and showed the results of descriptive analysis of those studies.

### Risk of bias assessment

Two reviewers in each group independently assessed the risk of bias of all included studies, and discrepancies were resolved by consensus. The risk of bias of the included randomized controlled trials was assessed using Cochrane’s risk of bias tool [24]. Potential sources of bias are examined according to six domains (including seven items): selection bias, performance bias, detection bias, attrition bias, reporting bias, and other biases. Each item was assessed as “low risk of bias,” “high risk of bias,” or “unclear.” The risk of bias of included non-randomized controlled trials was assessed using the tool of ROBINS-I [25], which contains seven items (confounding, selection of participants into the study, classification of the intervention, deviations from intended interventions, missing data, measurements of outcomes, and selections of the reported result), each of which was assessed as “low risk,” “moderate risk,” “serious risk,” “critical risk,” and “no information.” The Newcastle–Ottawa quality assessment scale [26, 27] was used to assess the risk of bias of cohort studies. The scale contains eight items in three domains: selection, comparability, and outcome. The items were rated with an asterisk. The Quality Appraisal Checklist for Case Series Studies developed by the Institute of Health Economics was used to assess the risk of bias of case series studies [28]. The checklist contains twenty items in eight domains: study objective, study population, intervention and co-intervention, outcome measure, statistical analysis, results and conclusions, competing interests and sources of support, and supplement. Each item was evaluated with “yes” or “no.”

### Data synthesis

A meta-analysis using the STATA14 software when the outcomes of included studies were highly consistent and descriptive analyses when there was high heterogeneity of outcomes between the included studies. According to Cochrane Handbook, when the meta-analysis was conducted, a random-effects meta-analysis for all outcomes was presented [29]. For an included study with intervention group and control group, the odds ratios (ORs) and their 95% confidence interval (CI) were used to describe the effect of dichotomous variables, while weighted mean differences (WMD) and their 95% CI were used to describe the effect of continuous variables. However, for an included study with only an intervention group, the effect sizes (ES) and their 95% CI were used to describe the effect of dichotomous variables while mean differences (MD) and their 95% CI were used to describe the effect of continuous variables. Statistical significance was set at < 0.05 on both sides [30]. We used the chi-squared test and *I*^2^ statistic were used to assess the level of statistical heterogeneity between the included studies, with *p* < 0.05 and *I*^2^ of less than 50% representing heterogeneity [30]. When substantial heterogeneity was detected, subgroup analyses by participant and study characteristics were used to compare pooled association estimates and heterogeneity. Furthermore, sensitivity analysis was used to detect potential outliers by omitting one estimate at a time and recalculating the pooled estimates. Publication bias was assessed through the funnel chart when the studies included in the meta-analyses were more than five [30].

### Quality of the evidence assessment

Two reviewers in each group independently assessed the quality of evidence using the grading of recommendations assessment, development, and evaluation (GRADE) approach for meta-analysis. We created a “Summary of findings” table using GRADEpro to show effect estimates derived from the body of evidence (quality of evidence) by outcome [31, 32]. Under the GRADE system, randomized controlled trials (RCTs) were initially assessed as high quality and observational studies as low quality. However, they were downgraded for reasons such as the risk of bias, inconsistency, indirectness, imprecision, publication bias, or upgraded for reasons such as the large magnitude of effect, dose–response gradient, and plausible confounding [33–38]. Thus, the quality of studies was rated as “high,” “medium,” “low,” and “very low,” reflecting the extent to which we are confident in the effect estimates.

Due to the peculiarity and public health significance of COVID-19, this study was not registered on the international registration platform PROSPERO.

## Results

### Study selection and characteristics

For clinical question 1, a total of 7292 records were retrieved from the databases and other methods. A total of two cohort studies were included, one was included from the database and another was unpublished studies obtained by data request [39, 40]. For clinical question 2, 8025 records were retrieved. A cohort [41] and case series [42] study was included by reading the title, abstract, and full text. For clinical question 3, 3657 records were retrieved, and six cohort studies [43–48] were finally included. The detailed screening process for each clinical question is shown in Supplementary File 3.

### Study characteristics

A total of 672 patients from the USA, Spain, France, UK, India, Serbia, and China were included in this study, of which the studies on IVIG were all from France (Table 1).

**Table 1:** Basic characteristics of the included studies

**Study**	**Region**	**Date**	**Study design**	**Total sample size**	**Sex (F/M)**	**Intervention**		**Control**		**Age, median (IQR), y**	**Drug**	
						Sample size	Details		Sample size	Details		
**Munoz et al. (2021) **[39]	America	Mar 6, 2021	Single-arm cohort	27	15/12	27	≥ 40 kg received RDV 200 mg IV Loading dose followed by RDV 100 mg IV daily for up to total of 10 day of treatment; < 40 kg received RDV 5 mg/kg loading dose followed by RDV 2.5 mg/kg IV daily for up to total of 10 days of treatment		NA	NA	10 (0.2–17)^b^	Remdesivir
**Goldman et al. (2021) **[40]	America	July 10, 2020	Single-arm cohort	77	31/46	77	≥ 40 kg received RDV 200 mg IV Loading dose followed by RDV 100 mg IV daily for up to total of 10 day of treatment; < 40 kg received RDV 5 mg/kg loading dose followed by RDV 2.5 mg/kg IV daily for up to total of 10 days of treatment		NA	NA	14 (0–17)^b^	Remdesivir
**García-Salido et al. (2020)**^**c**^ [41]	Spain	Nov 26, 2020	Cohort	61	23/38	40	Glucocorticoids (not specified)		21	No glucocorticoids	7.5 (4.9)^d^	Glucocorticoids
**Sun et al. (2020) **[42]	China	Mar 19, 2020	Case series	8	2/6	5	Glucocorticoids (not specified)		3	No glucocorticoids	6.8 (6.5)^d^	Glucocorticoids
**Ouldali et al. (2021)**^**a**^ [43]	France	Mar 2, 2021	Cohort	96	99/101	64	IVIG (2 g/kg) alone as first-line therapy		32	IVIG (2 g/kg) and methylprednisolone (0.8 to 1 mg/kg every 12 h (maximum of 30 mg for 12 h) for 5 days or a bolus of 15 to 30 mg/kg/d of methylprednisolone for 3 days.)	8.6 (4.7–12.1)	IVIG
**Belhadjer et al. (2020) **[44]	France	Dec 8, 2020	Cohort	40	NR	18	IVIG (2 g/kg) alone as first-line therapy		22	A combination of IVIG (2 g/kg once) and intravenous methylprednisolone (0.8 mg/kg/d for 5 days)	8.6 (6.7–11.2)	IVIG
**Son et al. (2021) **[45]	America	Jun 16, 2021	Cohort	206	NA	103	IVIG 2 g/kg (1.7, 2)		103	IVIG 2 g/kg (1.7, 2); methylprednisolone 2 mg/kg/day (1.5, 2.67) or Dexamethasone 0.3 mg/kg/day (0.15, 2) or Prednisolone 2 mg/kg/day (1, 2.1)	NA	IVIG
**McArdle et al. (2021) **[46]	United Kingdom	Jun 16, 2021	Cohort	420	NR	173	IVIG (not specified)		177 70	IVIG + glucocorticoids (not specified) Glucocorticoids (not specified)	NA	IVIG
**Sugunan et al. (2021) **[47]	India	Apr 20, 2021	Cohort	32	11/12	6	Children who were treated with IVIG received 2 g/kg as a continuous infusion over 8–12 h with longer duration in patients with cardiac dysfunction		26	Patients who were treated with methylprednisolone received pulse dose of 30 mg/kg once daily for 3 days followed by oral prednisolone at 2 mg/kg for 1 week or till CRP normalized, whichever was later. Steroid was tapered and stopped over the next 2–3 weeks	7.5 (5–9.5)	IVIG
**Vukomanovic et al. (2021) **[48]	Serbia	Jul 13, 2021	Cohort	22	7/15	10	Patients with mild-moderate form: Initially intravenous methylprednisolone (1 mg/kg) 5–7 days; After 5–7 days, oral prednisone (0.5–1 mg/kg) dose tapering on 5 days Patients with severe form without CAA: Initially intravenous methylprednisolone pulses (500 mg/m^2^) 3-day pulses + LMWHs in prophylactic doses; after the 3rd pulse intravenous methylprednisolone (1 mg/kg) 3–4 days + LMWHs in prophylactic doses; After 5–7 days, oral prednisone (0.5–1 mg/kg) dose tapering on 5 days		12	Patients with severe form with CAA complete Kawasaki disease: Initially intravenous immunoglobulins (1–2 g/kg) + intravenous methylprednisolone pulses (500 mg/m_2_) three-day pulses + LMWHs in prophylactic doses; after the 3rd pulse intravenous methylprednisolone (1 mg/kg) 3–4 days + LMWHs in prophylactic doses, After 5–7 days, oral prednisone (0.5–1 mg/kg) dose tapering on 5 days	9.3 (5.0)^d^	IVIG

*NA* not applicable, *NR* not report, *LMWH* low molecular weight heparin, *CAA* coronary artery disease

^a^After propensity score matching

^b^Mean (range)

^c^The data included in the meta-analysis of this article are not presented in the original article; we emailed the original author to ask for the data and get it

^d^Mean (standard deviation)

### Risk of bias assessment

The results of risk of bias are shown in Supplementary File 4. The GRADE quality summary of findings for all outcomes is shown in Supplementary File 5.

### Outcome of analysis

#### Remdesivir

One hundred and four patients in 2 single-arm cohort studies [39, 40] reported the efficacy and safety of remdesivir in treating children and adolescents with COVID-19. The results from a published study showed (*n* = 27) [39] that 22% of patients received mechanical ventilation and 26% received noninvasive ventilation or high-flow oxygen. In another study (*n* = 77) [40], all the patients were diagnosed with severe COVID-19, among which 50.6% were treated with mechanical ventilation and 26.0% with noninvasive ventilation or high-flow oxygen, 79% (61/77) of the patients had an underlying disease. The meta-analysis of 104 children and adolescents with COVID-19 who received remdesivir showed that 12.4% (95%CI, 6.1 to 18.8%, very low quality evidence) experienced obesity, 11.4% (95%CI, 3.5 to 19.4%, very low quality evidence) experienced asthma, 6.9% (95%CI, 0.0 to 19.4%, very low quality evidence) experienced immunosuppression/immunologic diseases, 13.3% (95%CI, 6.8 to 19.8%, very low quality evidence) experienced epilepsy, and 2.8% (95%CI, 0.0 to 6.0%, very low-quality evidence) experienced sickle cell disease.

The result of the meta-analysis showed that after the treatment, 54.7% (95%CI, 10.3 to 99.1%, very low-quality evidence) experienced adverse events, like acute kidney injury (19%, 5/27), constipation (15%, 4/27), increased alanine transaminase (ALT) (11%, 3/27), hyperglycemia (11%, 3/27), hypertension (11%, 3/27), pyrexia (11%, 3/27) [39], and anemia (3%, 2/77) [40]. There were 22.6% (95%CI, 5.6 to 39.6%, very low-quality evidence) of them experienced serious adverse events, 5.6% (95%CI, 1.2 to 10.1%) died, and 27.0% (95%CI, 0 to 73.0%, very low-quality evidence) needed ECMO or IMV.

#### Glucocorticoids

A retrospective cohort and case series studies [41, 42] comprising of 69 children or adolescents (age 7.41 ± 5.08) with severe COVID-19 treated with glucocorticoids were included. There was no statistically significant association between glucocorticoids therapy and mortality (OR = 2.79, 95% CI, 0.13 to 60.87, very low-quality evidence), mechanical ventilation rate (OR = 3.12, 95% CI, 0.80 to 12.08, very low-quality evidence), or the duration of PICU admission (WMD = 2.0, 95% CI, −0.95 to 4.95, very low-quality evidence).

##### IVIG

One cohort study [43] showed that 64 patients who received IVIG alone as first-line therapy had a treatment success rate of 62% (treatment failure defined as the persistence of fever 2 days after introducing first-line therapy or recrudescence of fever within 7 days after the first-line therapy). Patients with more severe initial clinical presentation (initial acute left ventricular dysfunction, initial PICU care, and hemodynamic support requirement) received a combination of IVIG and methylprednisolone as first-line therapy. The result showed that IVIG combined with methylprednisolone could decrease the treatment failure (OR = 0.25, 95%CI, 0.09 to 0.70, low-quality evidence), second-line treatment (OR = 0.19, 95%CI, 0.06 to 0.61, low-quality evidence), hemodynamic support (OR = 0.21, 95%CI, 0.06 to 0.76, low-quality evidence), the occurrence of secondary acute left ventricular dysfunction (OR = 0.20, 95%CI, 0.06 to 0.66, low-quality evidence), and duration of PICU stay (4 vs. 6, *p* = 0.005).

Another cohort study [44] included 22 MIS-C patients who received a combination of IVIG (2 g/kg) and methylprednisolone (0.8 mg/kg/d for 5d). They had a shorter recovery time from left ventricle ejection fraction (2.9 days vs 5.4 days, *p* = 0.002), isovolumic relaxation time (6.4 days vs 20.6 days, *p* < 0.0001), and duration of PICU stay (3.4 days vs 5.3 days, *p* < 0.05), in comparison with the 18 patients that received only IVIG (2 g/kg) as first-line therapy (Very low quality evidence).

Similarly, another cohort study [45] with larger sample size showed that IVIG plus glucocorticoids was associated with a lower risk of the composite outcome of cardiovascular dysfunction on or after day 2 than IVIG alone (17% vs. 31%; RR = 0.56, 95%CI, 0.34 to 0.94, very low quality evidence). The risks of the components of the composite outcome were also lower: left ventricular dysfunction (RR = 0.46, 95% CI, 0.19 to 1.15, very low quality evidence), shock resulting in vasopressor use (RR = 0.54, 95% CI, 0.29 to 1.00, very low quality evidence), and the use of adjunctive therapy (RR = 0.49, 95% CI, 0.36 to 0.65, very low quality evidence). However, in the other study [46] with 456 patients who met the WHO criteria for MIS-C, the authors compared IVIG plus glucocorticoids (*n* = 186) and glucocorticoids alone (*n* = 78) with IVIG alone (*n* = 246), and found modest evidence of benefit with glucocorticoids alone over IVIG. The primary outcomes were the receipt of inotropic support or mechanical ventilation on day 2 or later or death (IVIG plus glucocorticoids vs. IVIG: OR = 0.95, 95%CI: 0.37 to 2.45; glucocorticoids vs. IVIG: OR = 0.30, 95% CI: 0.10 to 0.85), and the reduction in the score for disease severity on the ordinal scale by day 2 (IVIG plus glucocorticoids vs. IVIG: OR = 1.09, 95% CI: 0.53 to 2.23; glucocorticoids vs. IVIG: OR = 1.95, 95% CI: 0.83 to 4.60) (very low quality evidence).

A cohort study [47] with 32 patients also compared glucocorticoids (*n* = 26) alone with IVIG alone (*n* = 6). Two patients each in glucocorticoids group and IVIG group failed treatment (very low quality evidence). Compared with IVIG, glucocorticoids group with a higher rate of c-reactive protein (CRP) levels less than 60 mg/L by day 3 (25% vs. 74%, *p* = 0.014, very low quality evidence).

For MIS-C patients with acute left ventricular systolic dysfunction or cardiogenic shock, a cohort study [48] (*n* = 22) showed that compared with the glucocorticoids group (*n* = 12), patients treated with IVIG plus glucocorticoids (3 days later, *n* = 10) with a higher prevalence of treatment failure (7/10 vs. 2/9, *p* = 0.03, very low quality evidence). ICU stays were shorter in the glucocorticoids group (4, IQR 2 to 5.5) than in the IVIG group (7, IQR 6 to 8.5) (*p* = 0.002, very low quality evidence).

## Discussion

### Key findings

A total of nine cohort studies and one case series study were included in this systematic review. For all of the three drugs, there were no large randomized controlled clinical trials performed in pediatric population in the context of COVID-19 or MIS-C has published, which may constitute an important gap of knowledge. In terms of remdesivir, there was no controlled study to prove its efficacy and safety in treating children and adolescents with COVID-19. Single-arm cohort studies have shown that the incidence of adverse reactions, mortality, and mechanical ventilation rate in patients treated with remdesivir are relatively low. As for glucocorticoids, the meta-analysis results showed no statistically significant difference in the improvement of mortality and mechanical ventilation rate between the intervention and control group. In terms of IVIG, most of the included cohort studies showed that for MIS-C patients with more severe clinical symptoms, IVIG combined with methylprednisolone could achieve better clinical efficacy than IVIG alone.

The use of remdesivir in COVID-19 patients is a controversial topic for both adults and children. A systematic review and network meta-analysis of adult patients based on randomized controlled trials showed that patients treated with remdesivir for 5 days had a higher rate of clinical improvement compared with placebo (OR = 1.68 (95% CI 1.18–12.40)). The rate of discharge (10-day remdesivir versus control: OR = 1.32 (95% CI 1.09–1.60); 5-day remdesivir versus control: OR = 1.73 (95% CI 1.28–2.35)) and recovery (10-day remdesivir versus control: OR = 1.29 (95% CI 1.03–1.60); 5-day remdesivir versus control: OR = 1.80 (95% CI 1.31–2.48)) of patients treated for 5 and 10 days were higher than placebo. Nevertheless, there was no significant improvement in mortality [49]. Although randomized clinical trials are the current golden standard for procuring evidence of a drug's efficacy, some authors have reported a greater benefit of the drug when it is used in a real-world population of patients with COVID-19 [50, 51], which were also worthy of attention. They suggest that the benefit could be even higher when it is used as the standard of care of patients with COVID-19. Based on this, on October 22, 2020, the US Food and Drug Administration (FDA) approved Veklury (remdesivir) for the treatment of COVID-19 in children and adolescents aged at least 12 years and weighing at least 40 kg requiring hospitalization [52]. It also approved an emergency use authorization of remdesivir to treat suspected or laboratory-confirmed COVID-19 in hospitalized pediatric patients weighing at least 3.5 kg but being either aged less than 12 years or weighing less than 40 kg [53]. The results of this systematic review showed that most of the children and adolescents included in this study had severe or underlying diseases, and the adverse events, mechanical ventilation rate, and mortality of the patients after treatment with remdesivir were low. Although there was a lack of control group in children’s studies, in adults’ clinical trials, the adverse effects were even higher in patients not treated with RDV compared to treated patients. Thus, high-quality clinical trials of children patients are needed. But the search in ClinicalTrials.gov showed that few studies focused only on children or adolescents with COVID-19 treated with remdesivir [54].

The effectiveness of glucocorticoids in the treatment of adult patients with COVID-19 has been confirmed. The Randomized Evaluation of COVID-19 Therapy (RECOVERY) Collaborative Group published an RCT on The New England Journal of Medicine, and the results of the study showed that among patients hospitalized with COVID-19, the use of dexamethasone resulted in lower 28-days mortality [9]. The WHO Rapid Evidence Appraisal for COVID-19 Therapies (REACT) working group published a systematic review based on seven RCTs. Results showed that systemic glucocorticoids administered to critically ill COVID-19 patients were associated with 28-day lower mortality than usual care or placebo [55]. Based on the systematic review evidence, the WHO developed a living guideline on glucocorticoids to recommend systemic glucocorticoids in treating patients with severe COVID-19 [12]. The recommendation was intended for the average patient population. However, the evidence that supported the recommendation was unclear for the under-represented population, such as children in the considered trials, which supported the meta-analysis of the systematic review. The search in ClinicalTrials.gov showed that no registered clinical trials have included or specifically targeted children or adolescents except for the RECOVERY trial. Most children with COVID-19 have only mild symptoms [5, 56], so it may be challenging to recruit critically ill children or adolescents to participate in clinical trials. The two studies included in this systematic review were observational studies with a small sample [41, 42] which found that glucocorticoids could not reduce the death rate in children or adolescents with critical COVID-19. Nevertheless, high-quality randomized controlled trials are recommended to confirm the effectiveness of glucocorticoids in the treatment of critically ill children or adolescents with COVID-19.

MIS-C is a unique complication in children and adolescents with COVID-19, which has similar characteristics to those of Kawasaki disease, but based on the limited evidence, the immunopathology of MIS-C remains a challenge [57]. Admittedly, IVIG generally produces anti-inflammatory effects, mitigates coronary artery abnormalities, and serves as first-line therapy of Kawasaki disease [13]. Several MIS-C guidelines are published, and the treatment therapy is based chiefly on Kawasaki disease, where IVIG is recommended empirically as the first-line treatment [54–56]. Besides, IVIG combined with glucocorticoids is also suggested as adjuvant therapy for severe patients or intensive therapy for patients with refractory diseases [58]. Three cohort studies included in this study showed that IVIG combined with glucocorticoids had better efficacy in MIS-C treatment than IVIG alone. Two of the three studies indicated that patients in the IVIG plus glucocorticoids group had more severe symptoms such as acute left ventricular dysfunction, admission to PICU care, and mechanical ventilation. The result is in agreement with the guideline recommendation of the use of IVIG in children and adolescents with COVID-19. Current guidelines also indicate a lack of high-quality studies comparing IVIG with glucocorticoids in MIS-C [58–61]. Different from the aforementioned three studies, two studies compared glucocorticoid-only with IVIG-only and the results provided modest evidence of benefit with glucocorticoids alone over IVIG alone [46, 47]. However, when expanding the range of patients to MIS-C and also those with any suspected inflammatory illness after SARS-CoV-2 infection, the data showed no differences between treatment with glucocorticoids or IVIG as single agents or between the single-agent and dual-agent treatments [46]. And among children with a myocardial injury during MIS-C, one cohort found that treatment with glucocorticoids only was associated with a faster normalization of fever, improved laboratory results, and a shorter ICU stay compared with IVIG-treated patients [48]. But the combination of IVIG and glucocorticoids is more suitable for severe children. The different results of these studies could be caused by different severity of diseases, the patient populations, and the time periods for which the investigators included the patients [62]. Although the four of the six cohort studies included in this study were of high quality, the results could not be combined due to the difference in their outcome indicators. The search in ClinicalTrials.gov showed that no study investigated the efficacy of IVIG as a therapeutic agent [63].

### Strengths and limitations

This study is the first systematic review accessing the remdesivir, glucocorticoids, and IVIG in treating children and adolescents with COVID-19. The study highlights the current status of evidence, identifies research gaps, and proffers recommendations for developing clinical practice guidelines in treating children and adolescents with COVID-19. However, there are also some limitations: (1) All the studies using remdesivir in treating children were low-quality single-arm cohort studies; thus, its efficacy and safety could not be clearly ascertained. (2) Due to the small sample size in the studies using glucocorticoids as treatment included in the study, the results of meta-analysis may be biased to some extent; however, fortunately, the small sample original research included did not take any weight of the meta-analysis. So, we, therefore, consider that this has little impact on the results of the analysis. And (3) quantitative analysis of studies on the treatment of MIS-C by IVIG was not feasible due to the heterogeneity of their outcome indicators.

### Further suggestions

Based on the results of this systematic review, we recommend (1) high-quality randomized controlled trials of potentially effective drugs for children with COVID-19 and (2) develop better guidelines based on substantial current evidence, provide a timely guide for clinical workers, and update them in real-time according to the evidence situation.

## Conclusion

Overall, the current evidence in the included studies is insignificant and of low quality, which does not adequately demonstrate the effectiveness and safety of using remdesivir, glucocorticoids, and IVIG in treating children and adolescents with COVID-19 or MIS-C. Therefore, it is recommended to conduct high-quality randomized controlled trials to provide substantial evidence for the development of guidelines.

## Supplementary Information

Below is the link to the electronic supplementary material.

Supplementary file1 (DOCX 37 kb)

Supplementary file2 (DOCX 40 kb)

Supplementary file3 (DOCX 528 kb)

Supplementary file4 (DOCX 23 kb)

Supplementary file5 (DOCX 44 kb)

## Data Availability

The datasets used and analyzed during this study are available from the corresponding author on reasonable request.

## Abbreviations

ALT: Alanine transaminase
CDC: Center for Disease Control and Prevention
CBM: China Biology Medicine
CNKI: China National Knowledge Infrastructure
CI: Confidence interval
COVID-19: Coronavirus disease 2019
ES: Effect sizes
ECMO: Extracorporeal membrane oxygenation
FDA: Food and Drug Administration
GRADE: Grading of Recommendations Assessment, Development, and Evaluation
IVIG: Intravenous immunoglobulin
IMV: Invasive mechanical ventilation
MD: Mean differences
ORs: Odds ratios
PICU: Pediatric intensive care unit
PRISMA: Preferred Reporting Items for Systematic Reviews and Meta Analyses
RCTs: Randomized controlled trials
RECOVERY: Randomized Evaluation of COVID-19 Therapy
REACT: Rapid Evidence Appraisal for COVID-19 Therapies
RNA: Ribonucleic acid
RCPCH: Royal College of Pediatrics and Child Health
SARS-CoV-2: Syndrome Coronavirus-2
WMD: Weighted mean differences
WHO: World Health Organization

## Declarations

This article is based on previously conducted studies and does not contain any studies with human participants or animals performed by any of the authors.

## References

[1] Horbach SPJM (2020) Pandemic publishing: medical journals strongly speed up their publication process for COVID-19. Quantitative Science Studies 1(3):1056–67

[2] Norris SL (2018). Meeting public health needs in emergencies-World Health Organization guidelines. J Evid Based Med.

[3] Wadaa-Allah A, Emhamed MS, Sadeq MA (2021). Efficacy of the current investigational drugs for the treatment of COVID-19: a scoping review. Ann Med.

[4] Wang Z, Zhou Q, Wang C (2020). Clinical characteristics of children with COVID-19: a rapid review and meta-analysis. Ann Transl Med.

[5] Shen K, Yang Y, Wang T (2020). Diagnosis, treatment, and prevention of 2019 novel coronavirus infection in children: experts' consensus statement. World J Pediatr.

[6] Wang H (2020) To investigate the application value of remdesivir in the treatment of COVID-19 patients. J Intern Intensive Med 26(06):513–5+528

[7] Siemieniuk R, Rochwerg B, Agoritsas T et al (2020) A living WHO guideline on drugs for covid-19. BMJ 370:m3379 10.1136/bmj.m3379 32887691

[8] Chiotos K, Hayes M, Kimberlin DW (2021). Multicenter interim guidance on use of antivirals for children with coronavirus disease 2019/severe acute Respiratory Syndrome Coronavirus 2. J Pediatric Infect Dis Soc.

[9] RECOVERY Collaborative Group, Horby P, Lim WS et al (2021) Dexamethasone in Hospitalized Patients with Covid-19. N Engl J Med 384(8):693–704 10.1056/NEJMoa2021436 PMC7383595 32678530

[10] Carsana L, Sonzogni A, Nasr A (2020). Pulmonary post-mortem findings in a series of COVID-19 cases from northern Italy: a two-centre descriptive study. Lancet Infect Dis.

[11] World Health Organization. Coronavirus disease (COVID-19): Dexamethasone. Available from: https://www.who.int/emergencies/diseases/novel-coronavirus-2019/question-and-answers-hub/q-a-detail/q-a-dexamethasone-and-covid-19. Accessed 5 Aug 2020

[12] World Health Organization. Corticosteroids for COVID-19: living guidance. Available from: https://apps.who.int/iris/handle/10665/334125. Accessed 5 Aug 2020

[13] McCrindle BW, Rowley AH, Newburger JW (2017). Diagnosis, treatment, and long-term management of kawasaki disease: a scientific statement for health professionals from the American Heart Association. Circulation.

[14] World Health Organization. Multisystem inflammatory syndrome in children and adolescents with COVID-19. Available from: https://www.who.int/publications/i/item/multisystem-inflammatory-syndrome-in-children-and-adolescents-with-covid-19. Accessed 5 Aug 2020

[15] Centers for Disease Control and Prevention. Emergency preparedness and response: health alert network. Available from: https://emergency.cdc.gov/han/2020/han00432.asp. Accessed 5 Aug 2020

[16] Royal College of Paediatrics and Child Health. Guidance: paediatric multisystem inflammatory syndrome temporally associated with COVID-19. Available from: https://www.rcpch.ac.uk/sites/default/files/2020-05/COVID-19-Paediatric-multisystem-%20inflammatory%20syndrome-20200501.pdf. Accessed 5 Aug 2020

[17] Centers for Disease Control and Prevention. Multisystem inflammatory syndrome (MIS-C). Available from: https://www.cdc.gov/mis-c/. Accessed 5 Aug 2020

[18] Hennon TR, Penque MD, Abdul-Aziz R et al (2020) COVID-19 associated multisystem inflammatory syndrome in children (MIS-C) guidelines: a Western New York approach. Prog Pediatr Cardiol 101232 10.1016/j.ppedcard.2020.101232 PMC7244417 32837142

[19] Page MJ, McKenzie JE, Bossuyt PM et al (2021) The PRISMA 2020 statement: an updated guideline for reporting systematic reviews. BMJ 372:n71. 10.1136/bmj.n71 10.1136/bmj.n71 PMC8005924 33782057

[20] World Health Organization. Multisystem inflammatory syndrome in children and adolescents with COVID-19. Available from: https://www.who.int/publications/i/item/multisystem-inflammatory-syndrome-in-children-and-adolescents-with-covid-19. Accessed 5 Aug 2020

[21] Centers for Disease Control and Prevention. Information for healthcare providers about multisystem inflammatory syndrome in children (MIS-C). Available from: https://www.cdc.gov/mis/mis-c/hcp/index.html. Accessed 5 Aug 2020

[22] Royal College of Paediatrics and Child Health. Guidance: Paediatric multisystem inflammatory syndrome temporally associated with COVID-19. London (UK): Royal College of Paediatrics and Child Health; 2020. Available at: www.rcpch.ac.uk/resources/guidance-paediatric-multisystem-inflammatory-syndrome-temporally-associated-covid-19. Accessed 5 Aug 2020

[23] Furukawa TA, Barbui C, Cipriani A, Brambilla P, Watanabe N (2006). Imputing missing standard deviations in meta-analyses can provide accurate results. J Clin Epidemiol.

[24] Higgins JPT, Altman DG, Gotzsche PC et al (2011) The Cochrane Collaboration’s tool for assessing risk of bias in randomised trials. BMJ 343:d5928 10.1136/bmj.d5928 PMC3196245 22008217

[25] Sterne JAC, Hernán MA, Reeves BC et al (2016) ROBINS-I: a tool for assessing risk of bias in non-randomised studies of interventions. BMJ 355:i4919 10.1136/bmj.i4919 PMC5062054 27733354

[26] Wells G, Shea B, O'Connell D et al. Newcastle– Ottawa Quality Assessment Scale—case control studies. Available from: http://www.ohri.ca/programs/clinical_epidemiology/oxford.asp. Accessed 5 Aug 2020

[27] Zhang Y, Huang L, Wang D, Ren P, Hong Q, Kang D (2021). The ROBINS-I and the NOS had similar reliability but differed in applicability: a random sampling observational studies of systematic reviews/meta-analysis. J Evid Based Med.

[28] Guo B, Moga C, Harstall C, Schopflocher D (2016). A principal component analysis is conducted for a case series quality appraisal checklist. J Clin Epidemiol.

[29] DerSimonian R, Kacker R (2007). Random-effects model for meta-analysis of clinical trials: an update. Contemp Clin Trials.

[30] Higgins JPT, Thomas J, Chandler J et al. Cochrane Handbook for Systematic Reviews of Interventions version 6.0 (updated July 2019). Cochrane. 2019. Available online: www.training.cochrane.org/handbook. Accessed 5 Aug 2020

[31] Guyatt G, Oxman AD, Akl EA et al (2011) GRADE guidelines: 1. Introduction—GRADE evidence profiles and summary of findings tables. J Clin Epidemiol 64(4):383–94 10.1016/j.jclinepi.2010.04.026 21195583

[32] GRADEpro GDT. GRADEpro Guideline Development Tool [Software]. McMaster University, 2015 (developed by Evidence Prime, Inc.). Available from: www.gradepro.org. Accessed 5 Aug 2020

[33] Guyatt GH, Oxman AD, Vist G et al (2011) GRADE guidelines: 4. Rating the quality of evidence—study limitations (risk of bias). J Clin Epidemiol 64(4):407–15 10.1016/j.jclinepi.2010.07.017 21247734

[34] Guyatt GH, Oxman AD, Montori V et al (2011) GRADE guidelines: 5. Rating the quality of evidence—publication bias. J Clin Epidemiol 64(12):1277–82 10.1016/j.jclinepi.2011.01.011 21802904

[35] Guyatt GH, Oxman AD, Kunz R et al (2011) GRADE guidelines 6. Rating the quality of evidence—imprecision. J Clin Epidemiol 64(12):1283–93 10.1016/j.jclinepi.2011.01.012 21839614

[36] Guyatt GH, Oxman AD, Kunz R et al (2011) GRADE guidelines: 7. Rating the quality of evidence—inconsistency. J Clin Epidemiol 64(12):1294–302 10.1016/j.jclinepi.2011.03.017 21803546

[37] Guyatt GH, Oxman AD, Kunz R et al (2011) GRADE guidelines: 8. Rating the quality of evidence—indirectness. J Clin Epidemiol 64(12):1303–10 10.1016/j.jclinepi.2011.04.014 21802903

[38] Guyatt GH, Oxman AD, Sultan S et al (2011) GRADE guidelines: 9. Rating up the quality of evidence. J Clin Epidemiol 64(12):1311–6 10.1016/j.jclinepi.2011.06.004 21802902

[39] Munoz F, Muller W, Ahmed A (2021). Safety and Efficacy of Remdesivir in a Pediatric COVID-19 Population. Virtual CROI.

[40] Goldman DL, Aldrich ML, Hagmann SHF et al (2021) Compassionate use of remdesivir in Children With Severe COVID-19. Pediatrics 147(5):e2020047803 10.1542/peds.2020-047803 33883243

[41] García-Salido A, de Carlos Vicente JC, Belda Hofheinz S (2020). Severe manifestations of SARS-CoV-2 in children and adolescents: from COVID-19 pneumonia to multisystem inflammatory syndrome: a multicentre study in pediatric intensive care units in Spain. Crit Care.

[42] Sun D, Li H, Lu XX (2020). Clinical features of severe pediatric patients with coronavirus disease 2019 in Wuhan: a single center's observational study. World J Pediatr.

[43] Ouldali N, Toubiana J, Antona D (2021). Association of intravenous immunoglobulins plus methylprednisolone vs immunoglobulins alone with course of fever in multisystem inflammatory syndrome in children. JAMA.

[44] Belhadjer Z, Auriau J, Méot M (2020). Addition of corticosteroids to immunoglobulins is associated with recovery of cardiac function in multi-inflammatory syndrome in children. Circulation.

[45] Son MBF, Murray N, Friedman K (2021). Multisystem inflammatory syndrome in children — initial therapy and outcomes. N Engl J Med.

[46] McArdle AJ, Vito O, Patel H (2021). Treatment of multisystem inflammatory syndrome in children. N Engl J Med.

[47] Sugunan S, Bindusha S, Geetha S (2021). Clinical profile and short-term outcome of children with SARS-CoV-2 related multisystem inflammatory syndrome (MIS-C) treated with pulse methylprednisolone. Indian Pediatr.

[48] Vukomanovic V, Krasic S, Prijic S (2021). Recent experience: corticosteroids as a first-line therapy in children with multisystem inflammatory syndrome and COVID-19-related myocardial damage. Pediatr Infect Dis J.

[49] Lai CC, Chen CH, Wang CY, Chen KH, Wang YH, Hsueh PR (2021) Clinical efficacy and safety of remdesivir in patients with COVID-19: a systematic review and network meta-analysis of randomized controlled trials. J Antimicrob Chemother dkab093 10.1093/jac/dkab093 PMC8083728 33758946

[50] Olender SA, Perez KK, Go AS et al (2020) Remdesivir for Severe COVID-19 versus a cohort receiving standard of care. Clin Infect Dis ciaa1041 10.1093/cid/ciaa1041 PMC7454434 32706859

[51] Frost MT, Jimenez-Solem E, Ankarfeldt MZ, Nyeland ME, Andreasen AH, Petersen TS (2020). The Adaptive COVID-19 Treatment Trial-1 (ACTT-1) in a real-world population: a comparative observational study. Crit Care.

[52] U.S. FOOD & DRUG ADMINISTATION. FDA approves first treatment for COVID-19. Available from: https://www.fda.gov/news-events/press-announcements/fda-approves-first-treatment-covid-19. Accessed 5 Aug 2020

[53] U.S. FOOD & DRUG ADMINISTATION. Veklury (remdesivir) EUA fact sheet for healthcare providers, updated 10/22/20. Available from: https://www.fda.gov/media/137566/download. Accessed 24 June 2020

[54] ClinicalTrials.gov. Available from: https://clinicaltrials.gov/ct2/results?cond=COVID-19&term=remdesivir&type=&rslt=&age_v=&age=0&gndr=&intr=&titles=&outc=&spons=&lead=&id=&cntry=&state=&city=&dist=&locn=&rsub=&strd_s=&strd_e=&prcd_s=&prcd_e=&sfpd_s=&sfpd_e=&rfpd_s=&rfpd_e=&lupd_s=&lupd_e=&sort=. Accessed 5 August 2020

[55] WHO Rapid Evidence Appraisal for COVID-19 Therapies (REACT) Working Group, Sterne JAC, Murthy S et al ( 2020) Association between administration of systemic corticosteroids and mortality among critically ill patients with COVID-19: a meta-analysis. JAMA 324(13):1330–41 10.1001/jama.2020.17023 PMC7489434 32876694

[56] Ding Y, Yan H, Guo W (2020). Clinical characteristics of children with COVID-19: a meta-analysis. Front Pediatr.

[57] Martinez OM, Bridges ND, Goldmuntz E, Pascual V (2020). The immune roadmap for understanding multi-system inflammatory syndrome in children: opportunities and challenges. Nat Med.

[58] Henderson LA, Canna SW, Friedman KG (2021). American College of Rheumatology clinical guidance for multisystem inflammatory syndrome in children associated with SARS-CoV-2 and hyperinflammation in pediatric COVID-19: version 2. Arthritis Rheumatol.

[59] Harwood R, Allin B, Jones CE (2021). A national consensus management pathway for paediatric inflammatory multisystem syndrome temporally associated with COVID-19 (PIMS-TS): results of a national Delphi process. Lancet Child Adolesc Health.

[60] American Academy of Pediatrics. Multisystem inflammatory syndrome in children (MIS-C) interim guidance. Available from: https://services.aap.org/en/pages/2019-novel-coronavirus-covid-19-infections/clinical-guidance/multisystem-inflammatory-syndrome-in-children-mis-c-interim-guidance/. Accessed 5 Aug 2020

[61] Cattalini M, Taddio A, Bracaglia C (2021). Childhood multisystem inflammatory syndrome associated with COVID-19 (MIS-C): a diagnostic and treatment guidance from the Rheumatology Study Group of the Italian Society of Pediatrics. Ital J Pediatr.

[62] DeBiasi RL (2021). Immunotherapy for MIS-C - IVIG, glucocorticoids, and biologics. N Engl J Med.

[63] ClinicalTrials.gov. Available from: https://clinicaltrials.gov/ct2/results?cond=MIS-C&term=&cntry=&state=&city=&dist=. Accessed 5 Aug 2020

