# Supplementary material for: Potentially effective drugs for the treatment of COVID-19 or MIS-C in children: a systematic review
Source: Eur J Pediatr. 2022 Feb 22;181(5):2135–46. doi: 10.1007/s00431-022-04388-w (PMC8861482; doi:10.1007/s00431-022-04388-w)
Supplement: Supplementary file 2 — Supplementary file2 (DOCX 40 kb) [file 431_2022_4388_MOESM2_ESM.docx]

**Supplementary File 1**

**Remdesivir**

**PubMed (N=918)**

1. "COVID-19"[Mesh]
2. "SARS-CoV-2"[Mesh]
3. "COVID-19"[Title/Abstract]
4. "SARS-COV-2"[Title/Abstract]
5. "SARS COV 2"[Title/Abstract]
6. "Novel coronavirus"[Title/Abstract]
7. "2019-novel coronavirus"[Title/Abstract]
8. "Coronavirus disease-19"[Title/Abstract]
9. "Coronavirus disease 19"[Title/Abstract]
10. "Coronavirus disease 2019"[Title/Abstract]
11. "COVID 19"[Title/Abstract]
12. "Novel CoV"[Title/Abstract]
13. "2019-nCoV"[Title/Abstract]
14. "2019 nCoV"[Title/Abstract]
15. "2019-CoV"[Title/Abstract]
16. OR/#1-15
17. "Remdesivir"[Supplementary Concept]
18. "GS-441524"[Supplementary Concept]
19. "Remdesivir"[Title/Abstract]
20. "Veklury"[Title/Abstract]
21. "GS-5734"[Title/Abstract]
22. OR/#17-#21
23. #16 AND #22
24. "adult*"[Title]
25. #23 NOT #24
26. #25 AND Filters applied: Humans

**WOS (n=1899)**

1. "COVID-19"[Topic]
2. "SARS-COV-2"[Topic]
3. "SARS COV 2"[Topic]
4. "Novel coronavirus"[Topic]
5. "2019-novel coronavirus"[Topic]
6. "Coronavirus disease-19"[Topic]
7. "Coronavirus disease 19"[Topic]
8. "Coronavirus disease 2019"[Topic]
9. "COVID 19"[Topic]
10. "Novel CoV"[Topic]
11. "2019-nCoV"[Topic]
12. "2019 nCoV"[Topic]
13. "2019-CoV"[Topic]
14. OR/#1-#13
15. "Remdesivir"[Topic]
16. "Veklury"[Topic]
17. "GS-5734"[Topic]
18. OR/#15-17
19. #14 AND #18
20. "adult*"[Title]
21. #19 NOT #20
22. #21 AND Publication Data: 2019-2021

limit 数据库: ( WOS )

**WHO COVID (N=2360)**

1. tw:(Remdesivir)
2. tw:(GS-441524)
3. tw:(Veklury)
4. OR/#1-3

**Cochrane (N=95)**

1. MeSH descriptor: [COVID-19] explode all trees
2. (COVID-19):ti,ab,kw
3. (SARS-COV-2):ti,ab,kw
4. ("SARS COV 2"):ti,ab,kw
5. ("Novel coronavirus"):ti,ab,kw
6. ("2019-novel coronavirus"):ti,ab,kw
7. ("COVID 19"):ti,ab,kw
8. (SARS-COV-2):ti,ab,kw
9. ("SARS COV 2"):ti,ab,kw
10. ("Novel coronavirus"):ti,ab,kw
11. ("2019-novel coronavirus"):ti,ab,kw
12. ("2019-nCoV"):ti,ab,kw
13. ("2019 nCoV"):ti,ab,kw
14. ("2019-CoV"):ti,ab,kw
15. OR/#1-14
16. (Remdesivir):ti,ab,kw
17. (Veklury):ti,ab,kw
18. (GS-5734):ti,ab,kw
19. OR/#16-18
20. (adult*):ti,ab,kw
21. #19 NOT #20

**CNKI (N=103)**

1. "新型冠状病毒"(主题)
2. "COVID-19"(主题)
3. "COVID 19"(主题)
4. "2019-nCoV"(主题)
5. "2019 nCoV"(主题)
6. "2019-CoV"(主题)
7. "2019 CoV"(主题)
8. "SARS-CoV-2"(主题)
9. "SARS COV 2"(主题)
10. "新冠肺炎"(主题)
11. OR/#1-10
12. "瑞德西韦"(主题)
13. "伦地西韦"(主题)
14. "韦如意"(主题)

**WanFang (N=67)**

1. 主题:(新型冠状病毒)
2. 主题:(COVID-19)
3. 主题:("COVID 19")
4. 主题:(2019-nCoV)
5. 主题:("2019 nCoV")
6. 主题:(2019-CoV)
7. 主题:("2019 CoV")
8. 主题:(SARS-CoV-2)
9. 主题:("SARS COV 2")
10. 主题:(新冠肺炎)
11. OR/#1-10
12. 主题:(瑞德西韦)
13. 主题:(伦地西韦)
14. 主题:(韦如意)
15. OR/#12-14
16. #11 AND #15

**CBM (N=115)**

1. "新冠肺炎"[常用字段:智能]
2. "新型冠状病毒"[常用字段:智能]
3. "COVID-19"[常用字段:智能]
4. "COVID 19"[常用字段:智能]
5. "2019-nCoV"[常用字段:智能]
6. "2019 nCoV "[常用字段:智能]
7. "2019-CoV"[常用字段:智能]
8. "2019 CoV "[常用字段:智能]
9. "SARS-CoV-2"[常用字段:智能]
10. "SARS COV 2"[常用字段:智能]
11. OR/#1-10
12. "瑞德西韦"[常用字段:智能]
13. "伦地西韦"[常用字段:智能]
14. "韦如意"[常用字段:智能]
15. OR/#12-14
16. #11 AND #15

**Corticosteroid**

**PubMed (N=1720)**

1. "COVID-19"[Mesh]
2. "SARS-CoV-2"[Mesh]
3. "COVID-19"[Title/Abstract]
4. "SARS-COV-2"[Title/Abstract]
5. "SARS COV 2"[Title/Abstract]
6. "Novel coronavirus"[Title/Abstract]
7. "2019-novel coronavirus"[Title/Abstract]
8. "Coronavirus disease-19"[Title/Abstract]
9. "Coronavirus disease 19"[Title/Abstract]
10. "Coronavirus disease 2019"[Title/Abstract]
11. "COVID 19"[Title/Abstract]
12. "Novel CoV"[Title/Abstract]
13. "2019-nCoV"[Title/Abstract]
14. "2019 nCoV"[Title/Abstract]
15. "2019-CoV"[Title/Abstract]
16. OR#1-#15
17. "Glucocorticoids"[ Mesh]
18. "Beclomethasone"[Title/Abstract]
19. "betamethasone valerate"[Mesh]
20. "Cortodoxone"[Title/Abstract]
21. "Dexamethasone"[Title/Abstract]
22. "Hydrocortisone"[Title/Abstract]
23. "Hydroxycorticosteroids"[Title/Abstract]
24. "methylprednisolone"[Mesh]
25. "adrenal cortex hormone*"[Title/Abstract]
26. "beclomethasone*"[Title/Abstract]
27. "beclometasone*"[Title/Abstract]
28. "betamethasone*"[Title/Abstract]
29. "betametasone*"[Title/Abstract]
30. "clobetasol*"[Title/Abstract]
31. "corticoid*"[Title/Abstract]
32. "corticosteroid*"[Title/Abstract]
33. "corticosterone*"[Title/Abstract]
34. "cortisone*"[Title/Abstract]
35. "cortodoxone*"[Title/Abstract]
36. "dexamethasone*"[Title/Abstract]
37. "dexametasone*"[Title/Abstract]
38. "glucocortico*"[Title/Abstract]
39. "hydrocortisone*"[Title/Abstract]
40. "hydroxycorticosteroid*"[Title/Abstract]
41. "hydroxypregnenolone*"[Title/Abstract]
42. "methylprednisolone*"[Title/Abstract]
43. "prednisolone*"[Title/Abstract]
44. "prednisone*"[Title/Abstract]
45. "pregnenedione*"[Title/Abstract]
46. "pregnenolone*"[Title/Abstract]
47. "tetrahydrocortisol*"[Title/Abstract]
48. "triamcinolone*"[Title/Abstract]
49. OR#17-#48
50. #16 AND #50
51. Filters applied: Humans

**WHO COVID-19 Database(N=2263)**

1. "Glucocorticoids"[Title, abstract, subject]
2. "Beclomethasone"[Title, abstract, subject]
3. "betamethasone valerate"[Mesh]
4. "Cortodoxone"[Title, abstract, subject]
5. "Dexamethasone"[Title, abstract, subject]
6. "Hydrocortisone"[Title, abstract, subject]
7. "Hydroxycorticosteroids"[Title, abstract, subject]
8. "methylprednisolone"[Mesh]
9. "adrenal cortex hormone*"[Title, abstract, subject]
10. "beclomethasone*"[Title, abstract, subject]
11. "beclometasone*"[Title, abstract, subject]
12. "betamethasone*"[Title, abstract, subject]
13. "betametasone*"[Title, abstract, subject]
14. "clobetasol*"[Title, abstract, subject]
15. "corticoid*"[Title, abstract, subject]
16. "corticosteroid*"[Title, abstract, subject]
17. "corticosterone*"[Title, abstract, subject]
18. "cortisone*"[Title, abstract, subject]
19. "cortodoxone*"[Title, abstract, subject]
20. "dexamethasone*"[Title, abstract, subject]
21. "dexametasone*"[Title, abstract, subject]
22. "glucocortico*"[Title, abstract, subject]
23. "hydrocortisone*"[Title, abstract, subject]
24. "hydroxycorticosteroid*"[Title, abstract, subject]
25. "hydroxypregnenolone*"[Title, abstract, subject]
26. "methylprednisolone*"[Title, abstract, subject]
27. "prednisolone*"[Title, abstract, subject]
28. "prednisone*"[Title, abstract, subject]
29. "pregnenedione*"[Title, abstract, subject]
30. "pregnenolone*"[Title, abstract, subject]
31. "tetrahydrocortisol*"[Title, abstract, subject]
32. "triamcinolone*"[Title, abstract, subject]
33. OR#1-#32
34. Filters applied: Humans

**Cochrane library (N=305)**

1. MeSH descriptor:[COVID-19]explode all trees
2. MeSH descriptor:[SARS-CoV-2]explode all trees
3. "COVID-19":ab,ti
4. "SARS-COV-2":ab,ti
5. "SARS COV 2":ab,ti
6. "Novel coronavirus":ab,ti
7. "2019-novel coronavirus":ab,ti
8. "Coronavirus disease-19":ab,ti
9. "Coronavirus disease 19":ab,ti
10. "Coronavirus disease 2019":ab,ti
11. "COVID 19":ab,ti
12. "Novel CoV":ab,ti
13. "2019-nCoV":ab,ti
14. "2019 nCoV":ab,ti
15. "2019-CoV":ab,ti
16. OR#1-#15
17. MeSH descriptor:[Glucocorticoids]explode all trees
18. "Beclomethasone":ab,ti
19. MeSH descriptor:[betamethasone valerate]explode all trees
20. "Cortodoxone":ab,ti
21. "Dexamethasone":ab,ti
22. "Hydrocortisone":ab,ti
23. "Hydroxycorticosteroids":ab,ti
24. MeSH descriptor:[methylprednisolone]explode all trees
25. "adrenal cortex hormone*":ab,ti
26. "beclomethasone*":ab,ti
27. "beclometasone*":ab,ti
28. "betamethasone*":ab,ti
29. "betametasone*":ab,ti
30. "clobetasol*":ab,ti
31. "corticoid*":ab,ti
32. "corticosteroid*":ab,ti
33. "corticosterone*":ab,ti
34. "cortisone*":ab,ti
35. "cortodoxone*":ab,ti
36. "dexamethasone*":ab,ti
37. "dexametasone*":ab,ti
38. "glucocortico*":ab,ti
39. "hydrocortisone*":ab,ti
40. "hydroxycorticosteroid*":ab,ti
41. "hydroxypregnenolone*":ab,ti
42. "methylprednisolone*":ab,ti
43. "prednisolone*":ab,ti
44. "prednisone*":ab,ti
45. "pregnenedione*":ab,ti
46. "pregnenolone*":ab,ti
47. "tetrahydrocortisol*":ab,ti
48. "triamcinolone*":ab,ti
49. OR#17-#48
50. #16 AND #49

**Web of Science (N=3242)**

1. TOPIC:"COVID-19"
2. TOPIC:"SARS-CoV-2"
3. TOPIC:"SARS COV 2"
4. TOPIC:"Novel coronavirus"
5. TOPIC:"2019-novel coronavirus"
6. TOPIC:"Coronavirus disease-19"
7. TOPIC:"Coronavirus disease 19"
8. TOPIC:"Coronavirus disease 2019"
9. TOPIC:"COVID 19"
10. TOPIC:"Novel CoV"
11. TOPIC:"2019-nCoV"
12. TOPIC:"2019 nCoV"
13. TOPIC:"2019-CoV"
14. TOPIC:OR#1-#13
15. TOPIC:"Glucocorticoids"
16. TOPIC:"Beclomethasone"
17. TOPIC:"betamethasone valerate"
18. TOPIC:"Cortodoxone"
19. TOPIC:"Dexamethasone"
20. TOPIC:"Hydrocortisone"
21. TOPIC:"Hydroxycorticosteroids"
22. TOPIC:"methylprednisolone"
23. TOPIC:"adrenal cortex hormone*"
24. TOPIC:"beclomethasone*"
25. TOPIC:"beclometasone*"
26. TOPIC:"betamethasone*"
27. TOPIC:"betametasone*"
28. TOPIC:"clobetasol*"
29. TOPIC:"corticoid*"
30. TOPIC:"corticosteroid*"
31. TOPIC:"corticosterone*"
32. TOPIC:"cortisone*"
33. TOPIC:"cortodoxone*"
34. TOPIC:"dexamethasone*"
35. TOPIC:"dexametasone*"
36. TOPIC:"glucocortico*"
37. TOPIC:"hydrocortisone*"
38. TOPIC:"hydroxycorticosteroid*"
39. TOPIC:"hydroxypregnenolone*"
40. TOPIC:"methylprednisolone*"
41. TOPIC:"prednisolone*"
42. TOPIC:"prednisone*"
43. TOPIC:"pregnenedione*"
44. TOPIC:"pregnenolone*"
45. TOPIC:"tetrahydrocortisol*"
46. TOPIC:"triamcinolone*"
47. TOPIC:OR#15-#46
48. TOPIC:#14 AND #47

**CNKI (N=88)**

1. "新型冠状病毒"(TOPIC)
2. "COVID-19"(TOPIC)
3. "COVID 19"(TOPIC)
4. "2019-nCoV"(TOPIC)
5. "2019 nCoV"(TOPIC)
6. "2019-CoV"(TOPIC)
7. "2019 CoV"(TOPIC)
8. "SARS-CoV-2"(TOPIC)
9. "SARS COV 2"(TOPIC)
10. "新冠肺炎"(TOPIC)
11. OR#1-#10
12. "糖皮质激素"(TOPIC)
13. "泼尼松"(TOPIC)
14. "强的松"(TOPIC)
15. "曲安奈德"(TOPIC)
16. "地塞米松"(TOPIC)
17. "可的松"(TOPIC)
18. "甲泼尼龙"(TOPIC)
19. "甲强龙"(TOPIC)
20. OR#12-#19
21. #11 AND #20
22. 限制：医药卫生科技

**CBM (N=262)**

1. "新型冠状病毒"[常用字段:智能]
2. "COVID-19"[常用字段:智能]
3. "COVID 19"[常用字段:智能]
4. "2019-nCoV"[常用字段:智能]
5. "2019 nCoV"[常用字段:智能]
6. "2019-CoV"[常用字段:智能]
7. "2019 CoV"[常用字段:智能]
8. "SARS-CoV-2"[常用字段:智能]
9. "SARS COV 2"[常用字段:智能]
10. "新冠肺炎"[常用字段:智能]
11. OR#1-#10
12. "糖皮质激素"[常用字段:智能]
13. "泼尼松"[常用字段:智能]
14. "强的松"[常用字段:智能]
15. "曲安奈德"[常用字段:智能]
16. "地塞米松"[常用字段:智能]
17. "可的松"[常用字段:智能]
18. "甲泼尼龙"[常用字段:智能]
19. "甲强龙"[常用字段:智能]
20. OR#12-#19
21. #11 AND #20

**WanFang (N=127)**

1. "新型冠状病毒"[TOPIC]
2. "COVID-19"[TOPIC]
3. "COVID 19"[TOPIC]
4. "2019-nCoV"[TOPIC]
5. "2019 nCoV"[TOPIC]
6. "2019-CoV"[TOPIC]
7. "2019 CoV"[TOPIC]
8. "SARS-CoV-2"[TOPIC]
9. "SARS COV 2"[TOPIC]
10. "新冠肺炎"[TOPIC]
11. OR#1-#10
12. "糖皮质激素"[TOPIC]
13. "泼尼松"[TOPIC]
14. "强的松"[TOPIC]
15. "曲安奈德"[TOPIC]
16. "地塞米松"[TOPIC]
17. "可的松"[TOPIC]
18. "甲泼尼龙"[TOPIC]
19. "甲强龙"[TOPIC]
20. OR#12-#19
21. #11 AND #20

**IVIG**

**PubMed (N=175)**

#1. "COVID-19" [Supplementary Concept]

#2. "severe acute respiratory syndrome coronavirus 2" [Supplementary Concept]

#3. "2019-CoV"[Title/Abstract]

#4. "2019-nCoV"[Title/Abstract]

#5. "Novel CoV"[Title/Abstract]

#6. "COVID19"[Title/Abstract]

#7. "coronavirus disease 2019"[Title/Abstract]

#8. "coronavirus disease-19"[Title/Abstract]

#9. "2019-novel coronavirus"[Title/Abstract]

#10. "Novel coronavirus"[Title/Abstract]

#11. "SARS-COV-2"[Title/Abstract]

#12. "COVID-19"[Title/Abstract]

#13. #1-#12/ OR

#14. "pediatric multisystem inflammatory disease, COVID-19 related" [Supplementary Concept]

#15. "Kawasaki disease"[Title/Abstract]

#16. "KD-like syndrome"[Title/Abstract]

#17. "KD-like disease"[Title/Abstract]

#18. "Kawasaki-like syndrome"[Title/Abstract]

#19. "Kawasaki-like disease"[Title/Abstract]

#20. "PMIS"[Title/Abstract]

#21. "PIMS"[Title/Abstract]

#22. "MIS-C"[Title/Abstract]

#23. "multisystem inflammatory syndrome in children"[Title/Abstract]

#24. #14-#23/ OR

#25. "Immunoglobulins, Intravenous" [Mesh]

#26. "gamma-Globulins" [Mesh]

#27. "Intravenous Immunoglobulin*" [Title/Abstract]

#28. "Intravenous IG" [Title/Abstract]

#29. "immune globulin*" [Title/Abstract]

#30. IVIG[Title/Abstract]

#31. "IV Immunoglobulin*" [Title/Abstract]

#32. "Intravenous Antibodies" [Title/Abstract]

#33. "gamma globulin*" [Title/Abstract]

#34. "gamma-globulin*" [Title/Abstract]

#35. "Flebogamma DIF" [Title/Abstract]

#36. Gamunex [Title/Abstract]

#37. "Globulin-N" [Title/Abstract]

#38. "Globulin N" [Title/Abstract]

#39. Intraglobin [Title/Abstract]

#40. Gammagard [Title/Abstract]

#41. Gamimune [Title/Abstract]

#42. Gamimmune [Title/Abstract]

#43. Privigen [Title/Abstract]

#44. Sandoglobulin [Title/Abstract]

#45. Venoglobulin [Title/Abstract]

#46. Iveegam [Title/Abstract]

#47. Endobulin [Title/Abstract]

#48. Gammonativ [Title/Abstract]

#49. #25-#48/OR

#50. #13 AND #24 AND #49

**Web of Science (N=100)**

#1. TOPIC: "COVID-19"

#2. TOPIC: "severe acute respiratory syndrome coronavirus 2"

#3. TOPIC: "2019-CoV"

#4. TOPIC: "2019-nCoV"

#5. TOPIC: "Novel CoV"

#6. TOPIC: "COVID19"

#7. TOPIC: "coronavirus disease 2019"

#8. TOPIC: "coronavirus disease-19"

#9. TOPIC: "2019-novel coronavirus"

#10. TOPIC: "Novel coronavirus"

#11. TOPIC: "SARS-COV-2"

#12. #1-#11/ OR

#13. TOPIC: "Kawasaki disease"

#14. TOPIC: "KD-like syndrome"

#15. TOPIC: "KD-like disease"

#16. TOPIC: "Kawasaki-like syndrome"

#17. TOPIC: "Kawasaki-like disease"

#18. TOPIC: "PMIS"

#19. TOPIC: "PIMS"

#20. TOPIC: "MIS-C"

#21. TOPIC: "multisystem inflammatory syndrome in children"

#22. #13-#21/ OR

#21. TOPIC: "Intravenous Immunoglobulin*"

#22. TOPIC: "Intravenous IG"

#23. TOPIC: "immune globulin*"

#24. TOPIC: "IVIG"

#25. TOPIC: "IV Immunoglobulin*"

#26. TOPIC: "Intravenous Antibodies"

#27. TOPIC: "gamma globulin*"

#28. TOPIC: "gamma-globulin*"

#29. TOPIC: "Flebogamma DIF"

#30. TOPIC: "Gamune"

#31. TOPIC: "Globulin-N"

#32. TOPIC: "Globulin N"

#33. TOPIC: "Intraglobin"

#34. TOPIC: "Gammagard"

#35. TOPIC: "Gamimune"

#36. TOPIC: "Gamimmune"

#37. TOPIC: "Privigen"

#38. TOPIC: "Sandoglobulin"

#39. TOPIC: "Venoglobulin"

#40. TOPIC: "Iveegam"

#41. TOPIC: "Endobulin"

#42. TOPIC: "Gammonativ"

#43. #21-#42/OR

#44. #12 AND #22 AND #43

**Cochrane (N=5)**

#1. MeSH descriptor: [COVID-19] explode all trees

#2. MeSH descriptor: [SARS-CoV-2] explode all trees

#3. "COVID-19":ti,ab,kw

#4. "SARS-COV-2":ti,ab,kw

#5. "Novel coronavirus":ti,ab,kw

#6. "2019-novel coronavirus":ti,ab,kw

#7. "Novel CoV":ti,ab,kw

#8. "2019-nCoV":ti,ab,kw

#9. "2019-CoV":ti,ab,kw

#10. "coronavirus disease-19":ti,ab,kw

#11. "coronavirus disease 2019":ti,ab,kw

#12. "severe acute respiratory syndrome coronavirus 2":ti,ab,kw

#13. "COVID 19":ti,ab,kw

#14. #1-#13/ OR

#15. "Kawasaki disease":ti,ab,kw

#16. "KD-like syndrome":ti,ab,kw

#17. "KD-like disease":ti,ab,kw

#18. "Kawasaki-like syndrome":ti,ab,kw

#19. "Kawasaki-like disease":ti,ab,kw

#20. "PMIS":ti,ab,kw

#21. "PIMS":ti,ab,kw

#22. "MIS-C":ti,ab,kw

#23. "multisystem inflammatory syndrome in children":ti,ab,kw

#24. #15-#23/ OR

#25. MeSH descriptor: [Immunoglobulins, Intravenous] explode all trees

#26. MeSH descriptor: [gamma-Globulins] explode all trees

#27. "Intravenous Immunoglobulin*" :ti,ab,kw

#28. "Intravenous IG" :ti,ab,kw

#29. "immune globulin*" :ti,ab,kw

#30. "IVIG":ti,ab,kw

#31. "IV Immunoglobulin*" :ti,ab,kw

#32. "Intravenous Antibodies" :ti,ab,kw

#33. "gamma globulin*" :ti,ab,kw

#34. "gamma-globulin*" :ti,ab,kw

#35. "Flebogamma DIF" :ti,ab,kw

#36. "Gamunex" :ti,ab,kw

#37. "Globulin-N" :ti,ab,kw

#38. "Globulin N" :ti,ab,kw

#39. "Intraglobin" :ti,ab,kw

#40. "Gammagard" :ti,ab,kw

#41. "Gamimune" :ti,ab,kw

#42. "Gamimmune" :ti,ab,kw

#43. "Privigen" :ti,ab,kw

#44. "Sandoglobulin" :ti,ab,kw

#45. "Venoglobulin" :ti,ab,kw

#46. "Iveegam" :ti,ab,kw

#47. "Endobulin" :ti,ab,kw

#48. "Gammonativ" :ti,ab,kw

#49. #25-#48/OR

#50. #14 AND #24 AND #49

**WHO COVID-19 Database (N=163)**

#1. 'severe acute respiratory syndrome coronavirus 2'[Mesh]

#2. 'coronavirus disease 2019'[Mesh]

#3. 'COVID-19'[Title, abstract, subject]

#4. 'SARS-COV-2'[Title, abstract, subject]

#5. 'novel coronavirus'[Title, abstract, subject]

#6. '2019-novel coronavirus'[Title, abstract, subject]

#7. 'coronavirus disease-19'[Title, abstract, subject]

#8. 'coronavirus disease 2019'[Title, abstract, subject]

#9. 'COVID 19'[Title, abstract, subject]

#10. 'novel cov'[Title, abstract, subject]

#11. '2019-ncov'[Title, abstract, subject]

#12. '2019-cov'[Title, abstract, subject]

#13. 'severe acute respiratory syndrome coronavirus 2'[Title, abstract, subject]

#14. #1-#13/ OR

#15. 'pediatric multisystem inflammatory syndrome'[Mesh]

#16. 'Kawasaki disease'[Title, abstract, subject]

#17. 'KD-like syndrome'[Title, abstract, subject]

#18. 'KD-like disease'[Title, abstract, subject]

#19. 'Kawasaki-like syndrome'[Title, abstract, subject]

#20. 'Kawasaki-like disease'[Title, abstract, subject]

#21. 'PMIS'[Title, abstract, subject]

#22. 'PIMS'[Title, abstract, subject]

#23. 'MIS-C'[Title, abstract, subject]

#24. 'multisystem inflammatory syndrome in children'[Title, abstract, subject]

#25. #15-#24/ OR

#26. 'Immunoglobulins'[Mesh]

#27. 'Intravenous Immunoglobulin*'[Title, abstract, subject]

#28. 'Intravenous IG'[Title, abstract, subject]

#29. 'immune globulin*'[Title, abstract, subject]

#30. 'IVIG'[Title, abstract, subject]

#31. 'IV Immunoglobulin*'[Title, abstract, subject]

#32. 'Intravenous Antibodies'[Title, abstract, subject]

#33. 'gamma globulin*'[Title, abstract, subject]

#34. 'gamma-globulin*'[Title, abstract, subject]

#35. 'Flebogamma DIF'[Title, abstract, subject]

#36. 'Gamunex'[Title, abstract, subject]

#37. 'Globulin-N'[Title, abstract, subject]

#38. 'Globulin N'[Title, abstract, subject]

#39. 'Intraglobin'[Title, abstract, subject]

#40. 'Gammagard'[Title, abstract, subject]

#41. 'Gamimune'[Title, abstract, subject]

#42. 'Gamimmune'[Title, abstract, subject]

#43. 'Privigen'[Title, abstract, subject]

#44. 'Sandoglobulin'[Title, abstract, subject]

#45. 'Venoglobulin'[Title, abstract, subject]

#46. 'Iveegam'[Title, abstract, subject]

#47. 'Endobulin'[Title, abstract, subject]

#48. 'Gammonativ'[Title, abstract, subject]

#49. #26-#48/OR

#50. #14 AND #25 AND #49

**CNKI (N=3)**

#1. "新型冠状病毒"[TOPIC]

#2. "COVID-19"[TOPIC]

#3. "COVID 19"[TOPIC]

#4. "2019-nCoV"[TOPIC]

#5. "2019-CoV"[TOPIC]

#6. "SARS-CoV-2"[TOPIC]

#7. #1-#6/ OR

#8. "儿童多系统炎症综合征"[TOPIC]

#9. "儿童多系统炎性综合征"[TOPIC]

#10. "儿科多系统炎症综合征"[TOPIC]

#11. "儿科多系统炎性综合征"[TOPIC]

#12. "川崎样疾病"[TOPIC]

#13. "川崎样综合征"[TOPIC]

#14. "川崎病"[TOPIC]

#15. "MIS-C"[TOPIC]

#16. #8-#15/ OR

#17. "丙种球蛋白"[主题]

#18. "静脉丙球"[主题]

#19. "免疫球蛋白"[主题]

#20. "IVIG"[主题]

#21. #17-#20/ OR

#22. #7 AND #16 AND #21

**WanFang (N=2)**

#1. 题名或关键词:("新型冠状病毒")

#2. 题名或关键词:("COVID-19")

#3. 题名或关键词:("COVID 19")

#4. 题名或关键词:(SARS-COV-2)

#5. 题名或关键词:(2019-nCoV)

#6. 题名或关键词:(2019-CoV)

#7. OR/#1-6

#8. 题名或关键词:("儿童多系统炎症综合征")

#9. 题名或关键词:("儿童多系统炎性综合征")

#10. 题名或关键词:("儿科多系统炎症综合征")

#11. 题名或关键词:( "儿科多系统炎性综合征")

#12. 题名或关键词:( "川崎病")

#13. 题名或关键词:( "川崎样疾病")

#14. 题名或关键词:( "川崎样综合征")

#15. 题名或关键词:( "MIS-C")

#16. OR/#8-15

#17. 题名或关键词:("丙种球蛋白")

#18. 题名或关键词:("静脉丙球")

#19. 题名或关键词:("IVIG")

#20. 题名或关键词:("免疫球蛋白")

#21. #17-#20/OR

#22. #7 AND #16 AND #21

**CBM (N=3)**

#1."新型冠状病毒"[常用字段:智能]

#2."COVID-19"[常用字段:智能]

#3. "COVID 19"[常用字段:智能]

#4. "2019-nCoV"[常用字段:智能]

#5. "2019-CoV"[常用字段:智能]

#6."SARS-CoV-2"[常用字段:智能]

#7. #1-#6/OR

#8."儿童多系统炎症综合征"[常用字段:智能]

#9."儿童多系统炎性综合征"[常用字段:智能]

#10."儿科多系统炎症综合征"[常用字段:智能]

#11."儿科多系统炎性综合征"[常用字段:智能]

#12."川崎样疾病"[常用字段:智能]

#13."川崎样综合征"[常用字段:智能]

#14."川崎病"[常用字段:智能

#15."MIS-C"[常用字段:智能]

#16. #8-#15/OR

#17. "丙种球蛋白"[常用字段:智能]

#18. "静脉丙球"[常用字段:智能]

#19. "免疫球蛋白"[常用字段:智能]

#20. "IVIG"[常用字段:智能]

#21. #17-#20/OR

#22. #7 AND #16 AND #21
