# Supplementary material for: Potentially effective drugs for the treatment of COVID-19 or MIS-C in children: a systematic review
Source: Eur J Pediatr. 2022 Feb 22;181(5):2135–46. doi: 10.1007/s00431-022-04388-w (PMC8861482; doi:10.1007/s00431-022-04388-w)

**Supplementary File 3 PRISMA 2020 flow diagram**

Clinical question 1 (remdesivir)


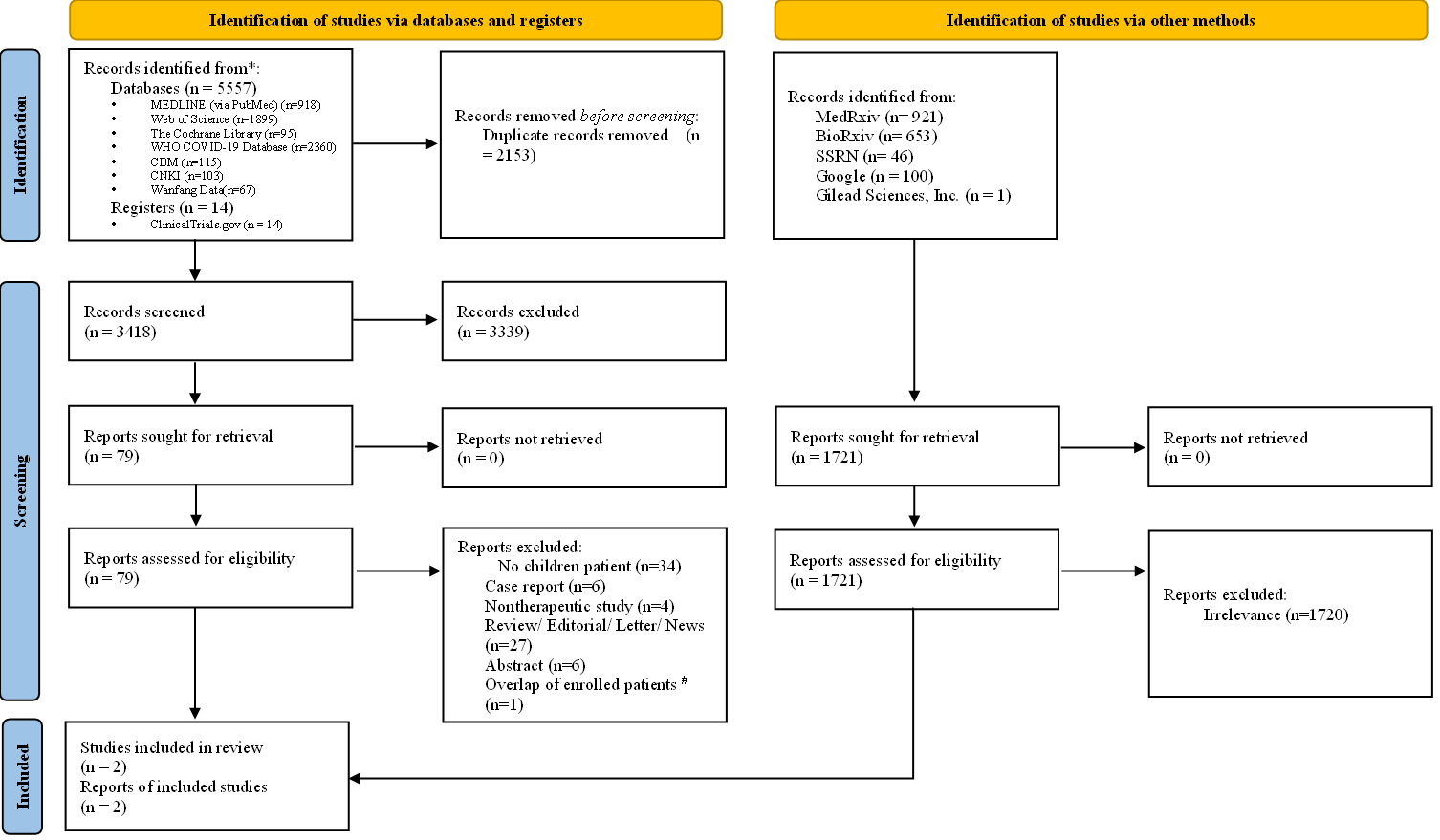


# Three patients in the cohort of Méndez-Echevarría et al. (Eur J Pediatr. 2021, n=8) were also included in Goldman et al. (Pediatrics. 2021, n=77) cohort. So we only include the Goldman cohort which with a larger sample size to improve the quality of the manuscript.

Clinical question 2 (glucocorticoids)


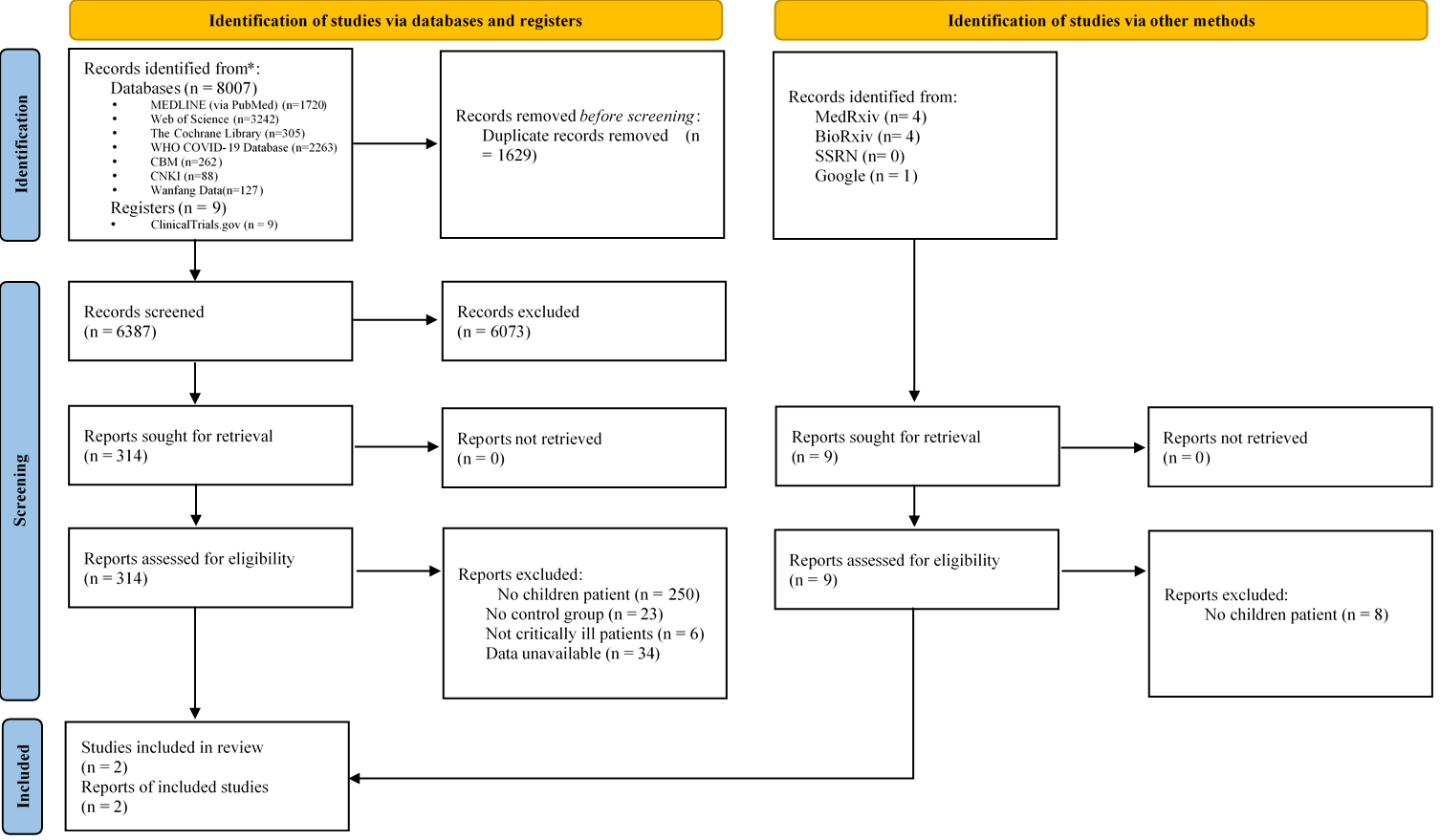


Clinical question 3 (IVIG)


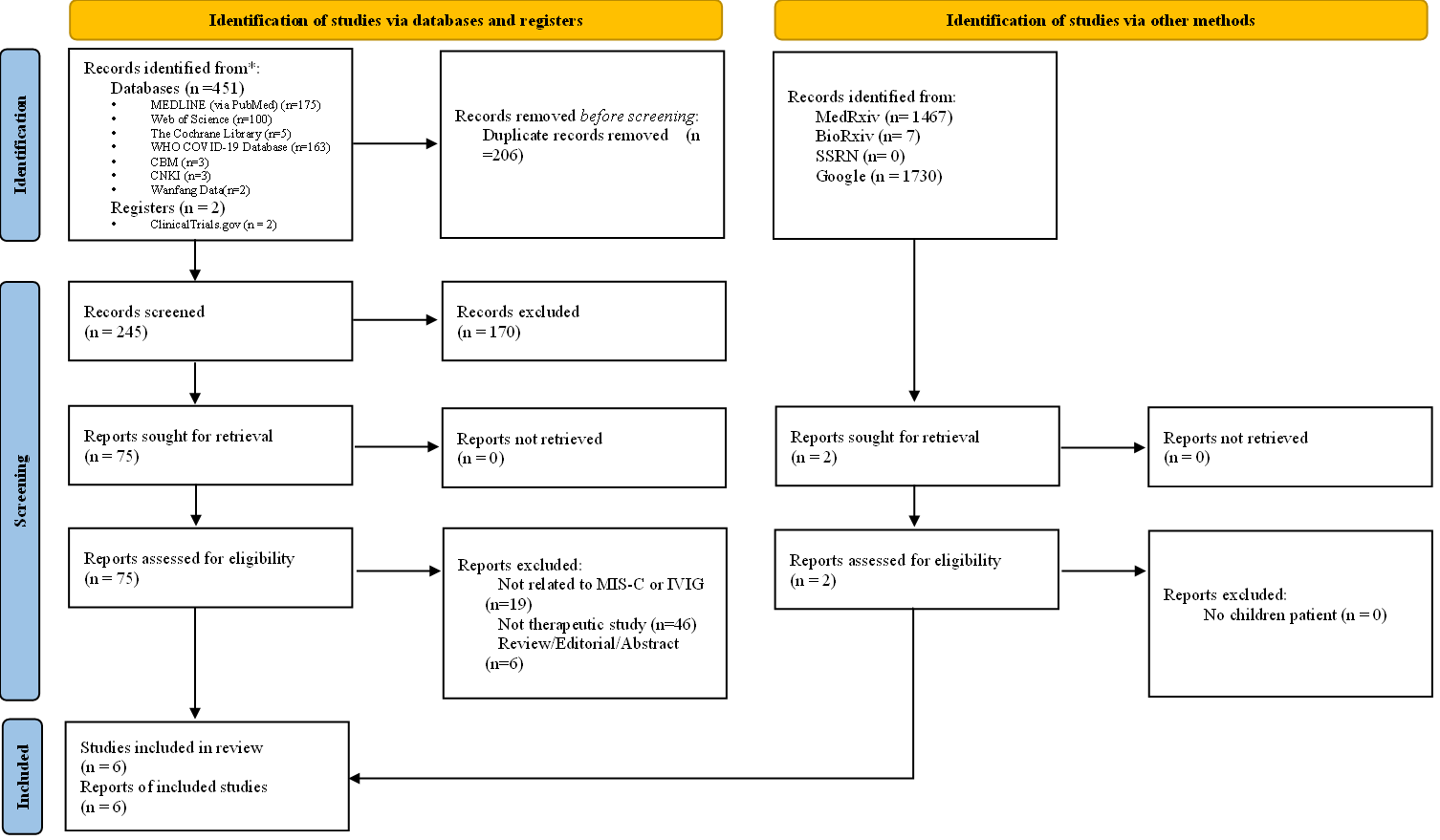

Supplement: Supplementary file 3 — Supplementary file3 (DOCX 528 kb) [file 431_2022_4388_MOESM3_ESM.docx]
