# Supplementary material for: Potentially effective drugs for the treatment of COVID-19 or MIS-C in children: a systematic review
Source: Eur J Pediatr. 2022 Feb 22;181(5):2135–46. doi: 10.1007/s00431-022-04388-w (PMC8861482; doi:10.1007/s00431-022-04388-w)
Supplement: Supplementary file 5 — Supplementary file5 (DOCX 44 kb) [file 431_2022_4388_MOESM5_ESM.docx]

**Supplementary File 4 GRADE assessment (Summary of Findings table)**

| Table 1. Remdesivir | | | | | | | | | |
| --- | --- | --- | --- | --- | --- | --- | --- | --- | --- |
| № of studies | Certainty assessment | | | | | № of patients | | Effect Value  (95% CI) | Certainty |
|  | Risk of bias | Inconsistency | Indirectness | Imprecision | Other considerations | Total | Event |  |  |
| Adverse events | | | | | | | | | |
| Single-arm Cohort (2) | Serious^1^ | Not serious | Not serious | Serious^2^ | None | 104 | 46 | 54.7% (10.3%, 99.1%) | ⨁◯◯◯  VERY LOW |
| Serious adverse events | | | | | | | | | |
| Single-arm Cohort (2) | Serious^1^ | Not serious | Not serious | Serious^2^ | None | 104 | 21 | 22.6% (5.6%, 39.6%) | ⨁◯◯◯  VERY LOW |
| Extra-corporeal membrane oxygenation (ECMO) or invasive mechanical ventilation (IMV) | | | | | | | | | |
| Single-arm Cohort (2) | Serious^1^ | Not serious | Not serious | Serious^2^ | None | 104 | 40 | 27.0% (0.0%, 73.0%) | ⨁◯◯◯  VERY LOW |
| Mortality | | | | | | | | | |
| Single-arm Cohort (2) | Serious^1^ | Not serious | Not serious | Serious^2^ | None | 104 | 6 | 5.6% (1.2%, 10.1%) | ⨁◯◯◯  VERY LOW |

| Table 2-1. Glucocorticoids | | | | | | | | | | |
| --- | --- | --- | --- | --- | --- | --- | --- | --- | --- | --- |
| № of studies | Certainty assessment | | | | | № of patients | | | Effect Value  (95% CI) | Certainty |
|  | Risk of bias | Inconsistency | Indirectness | Imprecision | Other considerations | Sample | Intervention | Control |  |  |
| Mortality | | | | | | | | | | |
| Cohort (1)  Case series (1) | Serious^1^ | Not serious | Not serious | Serious^2^ | None | 69 | 2/40 | 0/21 | 2.8 (0.1, 60.9) | ⨁◯◯◯  VERY LOW |
| Mechanical ventilation rate | | | | | | | | | | |
| Cohort (1)  Case series (1) | Serious^1^ | Not serious | Not serious | Serious^2^ | None | 69 | 14/40 | 3/21 | 3.1 (0.8, 12.1) | ⨁◯◯◯  VERY LOW |

| Table 2-2. Glucocorticoids | | | | | | | | | | | | |
| --- | --- | --- | --- | --- | --- | --- | --- | --- | --- | --- | --- | --- |
| № of studies | Certainty assessment | | | | | № of patients | | | | | Effect Value  (95% CI) | Certainty |
|  | Risk of bias | Inconsistency | Indirectness | Imprecision | Other considerations | Sample | Intervention | | Control | |  |  |
|  |  |  |  |  |  |  | Mean | SD | Mean | SD |  |  |
| Duration of PICU admission | | | | | | | | | | | | |
| Cohort (1) | Serious^1^ | Not serious | Not serious | Serious^2^ | None | 69 | 6.9 | 8.2 | 4.9 | 3.5 | 2.0 (-1.0, 5.0) | ⨁◯◯◯  VERY LOW |

| Table 3-1. IVIG (IVIG + Glucocorticoids vs. IVIG) | | | | | | | | | | |
| --- | --- | --- | --- | --- | --- | --- | --- | --- | --- | --- |
| № of studies | Certainty assessment | | | | | № of patients | | | Effect Value  (95% CI) | Certainty |
|  | Risk of bias | Inconsistency | Indirectness | Imprecision | Other considerations | Sample | Intervention | Control |  |  |
| Treatment failure | | | | | | | | | | |
| Cohort (1) | Serious^1^ | Not serious | Serious^3^ | Not serious | None | 96 | 3/32 | 24/64 | 0.3 (0.1, 0.7) | ⨁◯◯◯  VERY LOW |
| Second-line treatment | | | | | | | | | | |
| Cohort (1) | Serious^1^ | Not serious | Serious^3^ | Not serious | None | 96 | 3/32 | 20/64 | 0.2 (0.1, 0.6) | ⨁◯◯◯  VERY LOW |
| Hemodynamic support | | | | | | | | | | |
| Cohort (1) | Serious^1^ | Not serious | Serious^3^ | Not serious | None | 96 | 2/32 | 15/64 | 0.2 (0.1, 0.8) | ⨁◯◯◯  VERY LOW |
| LVEF <55% | | | | | | | | | | |
| Cohort (1) | Serious^1^ | Not serious | Serious^3^ | Not serious | None | 52 | 2/12 | 14/40 | 0.2 (0.1, 0.7) | ⨁◯◯◯  VERY LOW |

| Table 3-2. IVIG (IVIG + Glucocorticoids vs. IVIG) | | | | | | | | | | | | |
| --- | --- | --- | --- | --- | --- | --- | --- | --- | --- | --- | --- | --- |
| № of studies | Certainty assessment | | | | | № of patients | | | | | Effect Value  (95% CI) | Certainty |
|  | Risk of bias | Inconsistency | Indirectness | Imprecision | Other considerations | Sample | Intervention | | Control | |  |  |
|  |  |  |  |  |  |  | Mean | SD | Mean | SD |  |  |
| Duration of PICU admission | | | | | | | | | | | | |
| Cohort (1) | Serious^1^ | Not serious | Serious^3^ | Not serious | None | 96 | 3.6 | 2.3 | 6.1 | 3.4 | -2.4 (-4.0, -0.7) | ⨁◯◯◯  VERY LOW |
| Time to recovery of left ventricle ejection fraction | | | | | | | | | | | | |
| Cohort (1) | Serious^1^ | Not serious | Serious^3^ | Not serious | None | 22 | 2.9 | NR | 5.4 | NR | NR | ⨁◯◯◯  VERY LOW |
| Isovolumic relaxation time | | | | | | | | | | | | |
| Cohort (1) | Serious^1^ | Not serious | Serious^3^ | Not serious | None | 22 | 6.4 | NR | 20.6 | NR | NR | ⨁◯◯◯  VERY LOW |
| Duration of PICU stay | | | | | | | | | | | | |
| Cohort (1) | Serious^1^ | Not serious | Serious^3^ | Not serious | None | 22 | 3.4 | NR | 5.3 | NR | NR | ⨁◯◯◯  VERY LOW |

| Table 3-3. IVIG (IVIG + Glucocorticoids vs. IVIG) | | | | | | | | | | |
| --- | --- | --- | --- | --- | --- | --- | --- | --- | --- | --- |
| № of studies | Certainty assessment | | | | | № of patients | | | Effect Value  (95% CI) | Certainty |
|  | Risk of bias | Inconsistency | Indirectness | Imprecision | Other considerations | Sample | Intervention | Control |  |  |
| Cardiovascular dysfunction on or after day 2 | | | | | | | | | | |
| Cohort (1) | Serious^1^ | Not serious | Serious^3^ | Not serious | None | 206 | 18/103 | 32/103 | 0.6 (0.3, 0.9) | ⨁◯◯◯  VERY LOW |
| Left ventricular dysfunction | | | | | | | | | | |
| Cohort (1) | Serious^1^ | Not serious | Serious^3^ | Not serious | None | 150 | 6/75 | 13/75 | 0.5 (0.2, 1.2) | ⨁◯◯◯  VERY LOW |
| Shock resulting in vasopressor use | | | | | | | | | | |
| Cohort (1) | Serious^1^ | Not serious | Serious^3^ | Not serious | None | 204 | 13/102 | 24/102 | 0.5 (0.3, 1.0) | ⨁◯◯◯  VERY LOW |
| Use of adjunctive therapy | | | | | | | | | | |
| Cohort (1) | Serious^1^ | Not serious | Serious^3^ | Not serious | None | 212 | 36/106 | 74/106 | 0.5 (0.4, 0.7) | ⨁◯◯◯  VERY LOW |

| Table 3-4. IVIG (IVIG + Glucocorticoids vs. IVIG) | | | | | | | | | | |
| --- | --- | --- | --- | --- | --- | --- | --- | --- | --- | --- |
| № of studies | Certainty assessment | | | | | № of patients | | | Effect Value  (95% CI) | Certainty |
|  | Risk of bias | Inconsistency | Indirectness | Imprecision | Other considerations | Sample | Intervention | Control |  |  |
| Receipt of inotropic support or mechanical ventilation on day 2 or later or death | | | | | | | | | | |
| Cohort (1) | Serious^1^ | Not serious | Serious^3^ | Not serious | None | 331 | 54/162 | 40/169 | 1.0 (0.4, 2.5) | ⨁◯◯◯  VERY LOW |
| Reduction in the score for disease severity on the ordinal scale by day 2 | | | | | | | | | | |
| Cohort (1) | Serious^1^ | Not serious | Serious^3^ | Not serious | None | 304 | 52/152 | 43/152 | 1.1 (0.5, 2.2) | ⨁◯◯◯  VERY LOW |

| Table 3-4. IVIG (Glucocorticoids vs. IVIG) | | | | | | | | | | |
| --- | --- | --- | --- | --- | --- | --- | --- | --- | --- | --- |
| № of studies | Certainty assessment | | | | | № of patients | | | Effect Value  (95% CI) | Certainty |
|  | Risk of bias | Inconsistency | Indirectness | Imprecision | Other considerations | Sample | Intervention | Control |  |  |
| Receipt of inotropic support or mechanical ventilation on day 2 or later or death | | | | | | | | | | |
| Cohort (1) | Serious^1^ | Not serious | Serious^4^ | Not serious | None | 237 | 12/68 | 40/169 | 0.3 (0.1, 0.9) | ⨁◯◯◯  VERY LOW |
| Reduction in the score for disease severity on the ordinal scale by day 2 | | | | | | | | | | |
| Cohort (1) | Serious^1^ | Not serious | Serious^4^ | Not serious | None | 212 | 16/60 | 43/152 | 2.0 (0.8, 4.6) | ⨁◯◯◯  VERY LOW |

| Table 3-5. IVIG (IVIG vs. Glucocorticoids) | | | | | | | | | | |
| --- | --- | --- | --- | --- | --- | --- | --- | --- | --- | --- |
| № of studies | Certainty assessment | | | | | № of patients | | | Effect Value  (95% CI) | Certainty |
|  | Risk of bias | Inconsistency | Indirectness | Imprecision | Other considerations | Sample | Intervention | Control |  |  |
| Treatment failure | | | | | | | | | | |
| Cohort (1) | Serious^1^ | Not serious | Not serious | Serious^2^ | None | 32 | 2/6 | 2/26 | 4.3 (0.8, 24.9) | ⨁◯◯◯  VERY LOW |
| Rate of c-reactive protein (CRP) levels less than 60mg/L by day 3 | | | | | | | | | | |
| Cohort (1) | Serious^1^ | Not serious | Not serious | Serious^2^ | None | 32 | 2/6 | 17/26 | 0.5 (0.2, 1.6) | ⨁◯◯◯  VERY LOW |

| Table 3-6. IVIG (IVIG + Glucocorticoids vs. Glucocorticoids) | | | | | | | | | | |
| --- | --- | --- | --- | --- | --- | --- | --- | --- | --- | --- |
| № of studies | Certainty assessment | | | | | № of patients | | | Effect Value  (95% CI) | Certainty |
|  | Risk of bias | Inconsistency | Indirectness | Imprecision | Other considerations | Sample | Intervention | Control |  |  |
| Treatment failure | | | | | | | | | | |
| Cohort (1) | Serious^1^ | Not serious | Not serious | Serious^2^ | None | 19 | 7/10 | 2/9 | 3.2 (0.9, 11.4) | ⨁◯◯◯  VERY LOW |

| Table 3-6. IVIG (IVIG + Glucocorticoids vs. Glucocorticoids) | | | | | | | | | | | | |
| --- | --- | --- | --- | --- | --- | --- | --- | --- | --- | --- | --- | --- |
| № of studies | Certainty assessment | | | | | № of patients | | | | | Effect Value  (95% CI) | Certainty |
|  | Risk of bias | Inconsistency | Indirectness | Imprecision | Other considerations | Sample | Intervention | | Control | |  |  |
|  |  |  |  |  |  |  | Mean | SD | Mean | SD |  |  |
| Duration of PICU admission | | | | | | | | | | | | |
| Cohort (1) | Serious^1^ | Not serious | Not serious | Serious^2^ | None | 22 | 7.2 | 2.2 | 3.8 | 2.9 | 1.28 (0.4, 2.2) | ⨁◯◯◯  VERY LOW |

Explanations

1. downgrade one level: The risk of bias is high due to the limitations of study design

2. downgrade one level: Sample size is less than optimal information sample (OIS) or confidence interval is too wide

3. downgrade one level: Glucocorticoids combined with IVIG

4. downgrade one level: Glucocorticoids vs. IVIG

CI: Confidence interval; NR: Not report;
